# Prediction of the Effects of Synonymous Variants on SARS-CoV-2 Genome

**DOI:** 10.12688/f1000research.72896.1

**Published:** 2021-10-18

**Authors:** Wan Xin Boon, Boon Zhan Sia, Chong Han Ng

**Affiliations:** 1 Faculty of Information Science and Technology, Multimedia University, Bukit Beruang, Melaka, 75450, Malaysia

**Keywords:** COVID-19, SARS-CoV-2, Synonymous mutations, RNA secondary structure

## Abstract

**Background:** The emergence of severe acute respiratory syndrome coronavirus 2 (SARS-CoV-2) had led to a global pandemic since December 2019. SARS-CoV-2 is a single-stranded RNA virus, which mutates at a higher rate. Multiple studies had been done to identify and study nonsynonymous mutations, which change amino acid residues of SARS-CoV-2 proteins. On the other hand, there is little study on the effects of SARS-CoV-2 synonymous mutations. Although these mutations do not alter amino acids, some studies suggest that they may affect viral fitness. This study aims to predict the effect of synonymous mutations on the SARS-CoV-2 genome.

**Methods:** A total of 30,229 SARS-CoV-2 genomic sequences were retrieved from Global Initiative on Sharing all Influenza Data (GISAID) database and aligned using MAFFT. Then, the mutations and their respective frequency were identified. A prediction of RNA secondary structures and their base pair probabilities was performed to study the effect of synonymous mutations on RNA structure and stability. Relative synonymous codon usage (RSCU) analysis was also performed to measure the codon usage bias (CUB) of SARS-CoV-2.

**Results:** A total of 150 synonymous mutations were identified. The synonymous mutation identified with the highest frequency is C3037U mutation in the nsp3 of ORF1a, followed by C313U and C9286U mutation in nsp1 and nsp4 of ORF1a, respectively.

**Conclusion:** Among the synonymous mutations identified, C913U mutation in ORF1a and C26735U in membrane (M) protein may affect RNA secondary structure, reducing the stability of RNA folding and possibly resulting in a higher translation rate. However, lab experiments are required to validate the results obtained from prediction analysis.

## Introduction

In December 2019, coronavirus disease 2019 (COVID-19) cases first emerged from Wuhan, China ^1^. Soon after, rapid spread of COVID-19 has resulted in a serious global outbreak. COVID-19 is an infectious and potentially lethal disease caused by a newly found coronavirus strain, known as severe acute respiratory syndrome coronavirus 2 (SARS-CoV-2). The virus causes clinical manifestation ranging from asymptomatic to severe pneumonia and eventually death ^2^. SARS-CoV-2 seems to have a higher transmission rate ^3^ but lower mortality rate ^2^ in comparison to Middle East respiratory coronavirus (MERS-CoV) and severe acute respiratory syndrome coronavirus (SARS-CoV).

SARS-CoV-2 is a single-stranded RNA virus with a genome size of 29,903 bases. In general, RNA viruses have a higher mutation rate than DNA viruses and this allows them to evolve rapidly, escaping the host immune defence response ^4^. A study by Kim *et al*. (2020) identified a total of 1,352 nonsynonymous and 767 synonymous mutations from 4,254 SARS-CoV-2 genomes ^5^. While in another study done by Khailany *et al.* (2020), 116 mutations had been identified from 95 SARS-CoV-2 genomes ^6^.

In this study, we focus on synonymous mutations instead of nonsynonymous mutations as researchers often overlook their biological importance. Synonymous mutations are also known as silent mutations because the nucleotide mutations result in a change in the RNA sequence without altering the amino acid sequence ^7^. Synonymous mutations have been suggested to have no functional consequence on the fitness of organisms and their evolution in long term ^8^. However, numerous recent studies had showed that synonymous mutations may affect the folding and stability of RNA structures ^9^. For RNA viruses, even though synonymous mutations generally do not change their pathogenicity, some studies reveal that synonymous mutations may affect the RNA secondary structure of the virus ^10^ and also change the codon usage bias of the genes in the virus ^11, 12^.

## Methods

### Sequence retrieval

30,229 SARS-CoV-2 genomic sequences were retrieved from GISAID database (Global Initiative on Sharing All Influenza Data, RRID:SCR_018251) ^13^ ranging from 31 December 2019 to 22 March 2021. SARS-CoV-2 genomic sequences were filtered by setting parameters to keep only sequences with complete genome and high coverage. The reference sequence of SARS-CoV-2 genome ( NC_045512.2) ^14^ was retrieved in fasta format from NCBI database (NCBI, RRID:SCR_006472). It is a Wuhan isolate with a complete genome which comprises of 29,903 bases.

### Multiple sequence alignment

The rapid calculation available in MAFFT online server (MAFFT, version 7.467, RRID:SCR_011811) ^15^ was used to perform multiple sequence alignment (MSA) for 30,229 SARS-CoV-2 genomes. This option supports the alignment of more than 20,000 sequences with approximately 30,000 sites. The alignment length was kept, which means the insertions at the mutated sequences were removed, to keep the alignment length the same as the reference sequence. While other parameters were left as default.

### Identification of mutations and their frequency in SARS-CoV-2 genomes

A simple Python script was written to identify the mutations in 30,229 SARS-CoV-2 genomes. To determine whether the identified mutations are synonymous or nonsynonymous, MEGA X software, version 10.2.5 build 10210330 ( MEGA Software, RRID:SCR_000667) ^16^ was utilized to perform the translation for inspection purposes. The presence of amino acid changes was identified by referring to the genomic position of the nucleotide mutations. Synonymous mutations with the top 10 highest frequencies were generated.

### SARS-CoV-2 RNA secondary structure prediction

The RNA secondary structure of wild type and mutant sequences were predicted using RNAfold program, version 2.4.18 (Vienna RNA, RRID:SCR_008550) to show how mutations affect RNA secondary structure. The RNA secondary structure prediction was performed using a sequence length of 250 nucleotides upstream and downstream of the mutation site. The minimum free energy (MFE) was also calculated in the RNAfold program for both wild type and mutant sequences to show comparison of the RNA folding stability between them. The comparison on the value of minimum free energy (MFE) is important to indicate whether the mutations affect the folding stability of the respective RNA structure.

### Base pair probability estimation and analysis

To predict how the mutations affect RNA local folding, base pair probability was estimated by utilizing MutaRNA, version 1.3.0 (MutaRNA, RRID:SCR_021723) ^17^. MutaRNA is a web-based tool that allows prediction and visualization of the structure changes induced by a single nucleotide polymorphism (SNP) in an RNA sequence. It includes the base pair probabilities within RNA molecule of both wild type and mutant. The parameters used in MutaRNA were set as default, in which the window size is 200nt and the maximal base pair span is 150nt. 

### Relative synonymous codon usage (RSCU)

Relative synonymous codon usage (RSCU) represents the ratio of the observed frequency of codons appearing in a gene to the expected frequency under equal codon usage. RSCU is calculated using the formula:

$$RSCU_{i}=\frac{X_{i}}{\frac{1}{n}\sum_{i=1}^{n}X_{i}},$$

where X _i_ implies the number of occurrences of codon i and n stands for the number of synonymous codons encoded for that particular amino acid.

## Results and discussion

A synonymous mutation is a change in the nucleotide that does not cause any changes in the encoded amino acid. Synonymous mutations were previously considered to be less important, but they are now proven to have some effects on RNA folding, RNA stability, miRNA binding and translational efficiency ^18^. Synonymous mutations may have significant effects on the adaptation, virulence, and evolution of RNA viruses ^19^. Another study done also indicated that synonymous mutations have association with more than 50 human diseases such as hemophilia B, tuberculosis (TB), cystic fibrosis (CF), Alzheimer, schizophrenia, chronic hepatitis C and so on ^20^. All these studies show that increasing importance has been associated with synonymous mutations over these years. Hence, it is necessary for us to study the effects of synonymous mutations of SARS-CoV-2 genome.

### Identification of SARS-CoV-2 synonymous mutations

**Figure 1.**
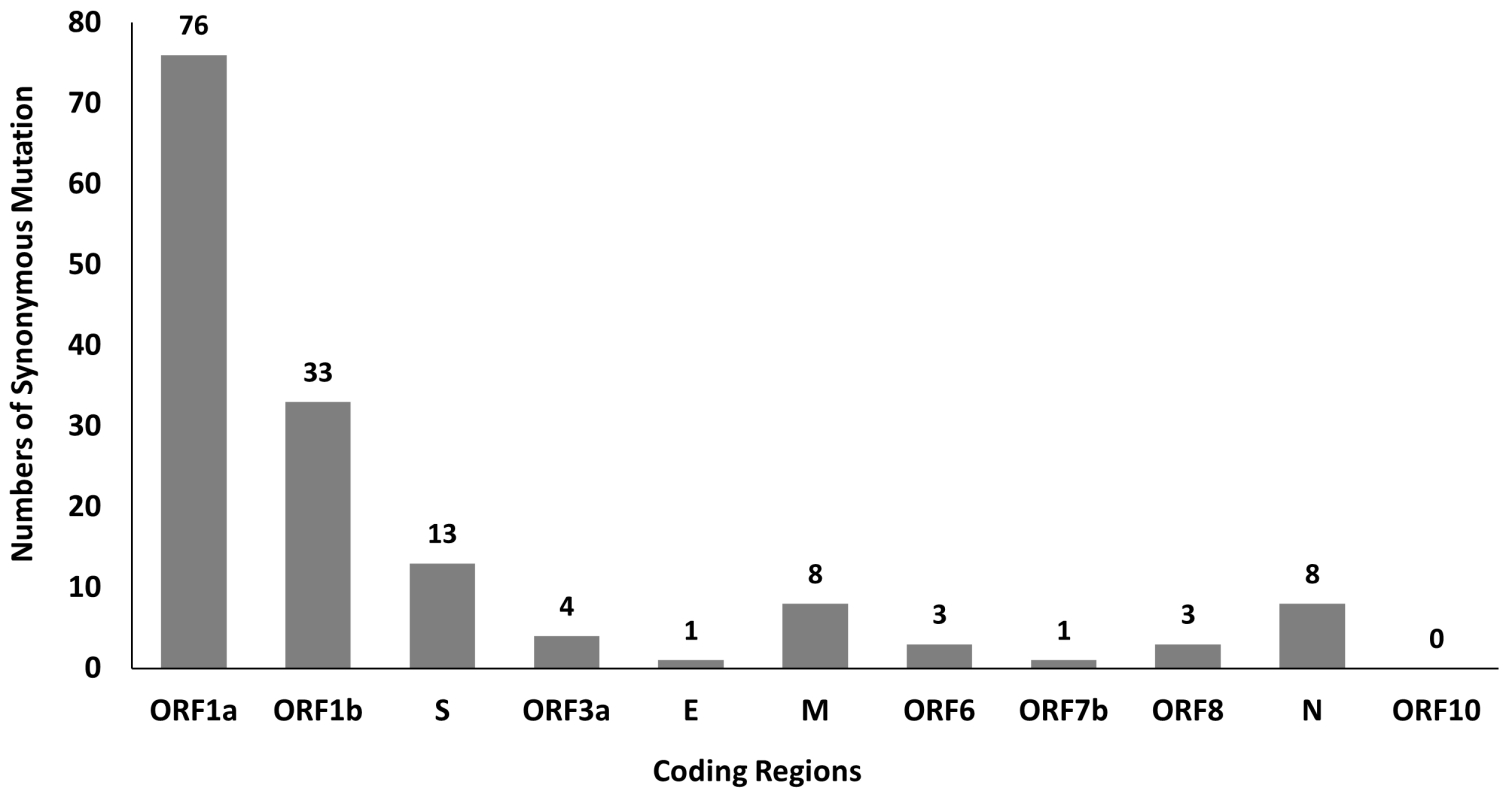
Distribution of SARS-CoV-2 synonymous mutations in 11 coding regions.

A total of 381 mutations were found in SARS-CoV-2 genomes by using python script, in which 150 of them are synonymous mutations. The distribution of synonymous mutations in 11 coding regions is shown in Figure 1. Among these mutations, ORF1a and ORF1b have a higher number of synonymous mutations at 76 and 33, respectively, which might be due to their longer sequence length. Besides that, our findings also show high C to U mutation rate in SARS-CoV-2 genetic variation and this mutational skews are in line with the studies done by Rice *et al.* (2021) and Simmonds (2020) ^21, 22^. These mutational skews are necessary to be considered when deducing the selection acting on synonymous variants in SARS-CoV-2 evolution ^23^.

 The synonymous mutations in SARS-CoV-2 genomes with the top 10 highest frequency were listed in Table 1. As shown in Table 1, synonymous mutations with the highest frequency identified from SARS-CoV-2 genomes is C3037U mutation located in nsp3 of ORF1a, followed by C313U mutation in nsp1 of ORF1a and C9286U mutation in nsp4 of ORF1a. Mutations with higher frequency are mostly found in ORF1a and ORF1b. It is of great interest to find out the effect of these top 10 synonymous mutations on SARS-CoV-2 genome.

**Table 1.** SARS-CoV-2 synonymous mutations with the top 10 highest frequency.

ORF		Position	Nucleotide Variation	Frequency
1a	nsp1	313	C > U	8569
	nsp2	913	C > U	3213
	nsp3	3037	C > U	28054
		5986	C > U	3261
	nsp4	9286	C > U	4030
1b	nsp12	14676	C > U	3262
		15279	C > U	3244
		16176	U > C	3236
	nsp14	18877	C > U	2944
M		26735	C > U	2701

### RNA secondary structure and base pair probability

SARS-CoV-2 virus can form highly structured RNA elements, which may affect viral replication, discontinuous transcription and translation ^24– 28^

The RNA secondary structures of wild type and mutant sequences were predicted using RNAfold program. Minimum free energy (MFE) of each structure was also calculated to show the folding stability of the respective RNA structure. Among all, C913U mutation and C26735U mutation were found to have a more obvious effect on the predicted RNA secondary structure compared to the wild type.

**Figure 2.**
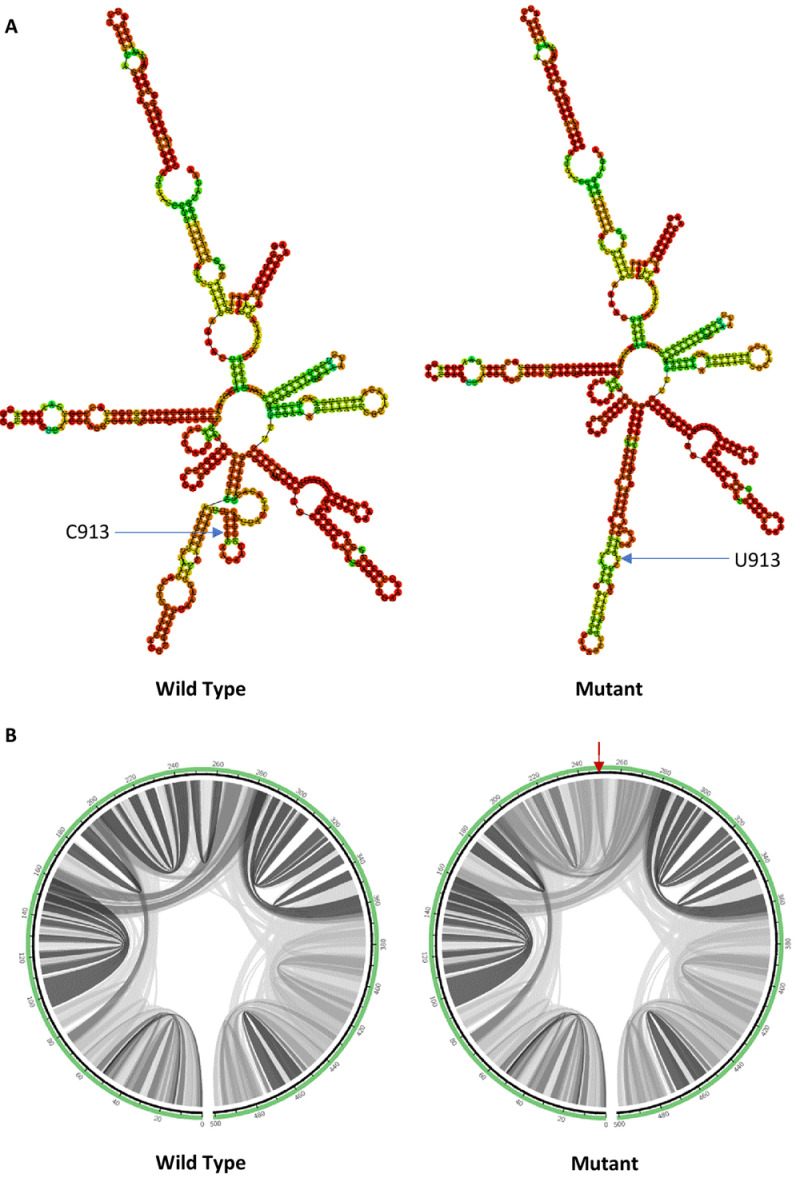
The effect of C913U mutation on RNA secondary structure of nsp2 in ORF1a. ( **A**) RNA secondary structure of wild type C913 and mutant U913. ( **B**) Circular plots showing the base pairing probabilities with darker shades indicating higher base pairing probabilities and a red arrow pointing to the mutation site.

As shown in Figure 2(A), a single hairpin is formed around the mutant with C913U mutation instead of a multiloop in the wild type. The minimum free energy value of the mutant (- 146.90 kcal/mol) is slightly less negative than that of the wild type (- 147.40 kcal/mol), which makes it a less thermodynamically stable structure compared to the wild type.

To visualize the differences in the base pairing potential induced by the mutation, base pairing probability was estimated using MutaRNA. The circular plots in Figure 2(B) show the base pairing probabilities of both wild type and mutant. As shown in the circular plots, C913U mutation decreased the Watson–Crick base pairing probability near the mutation site, which led to a less stable predicted RNA secondary structure. C913U mutation is found in the nsp2 of ORF1a in SARS-CoV-2 genome. Nsp2 in SARS-CoV interacts with two human host proteins specifically, which are prohibitin 1 (PHB1) and prohibitin 2 (PHB2) ^29^. This interaction may disrupt the intracellular host signalling during the viral infections, rather than taking part in the viral replication ^29^. It is yet to see if nsp2 of SARS-CoV-2 shares same or similar function as that of SARS-CoV.

**Figure 3.**
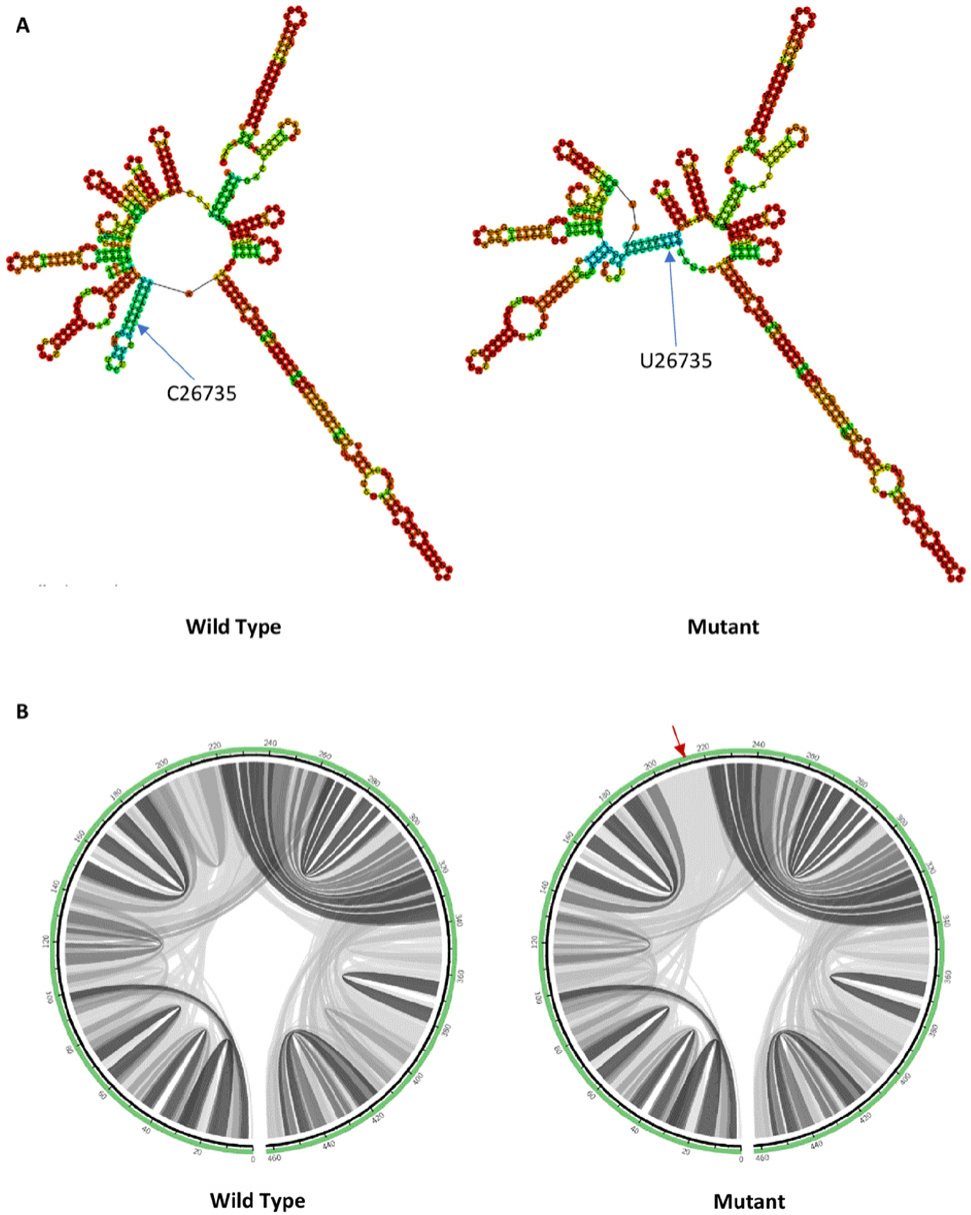
The effect of C26735U mutation on RNA secondary structure of M protein. ( **A**) RNA secondary structure of wild type C26735 and mutant U26735. ( **B**) Circular plots showing the base pairing probabilities with darker shades indicating higher base pairing probabilities and a red arrow pointing to the mutation site.

 C26735U mutation is located at the membrane (M) protein. M protein may suppress host immune responses via the interference of Type I interferon production ^30^. C26735U mutation induces changes in the predicted RNA secondary structure by forming an extra multibranch loop at the mutation site as shown in Figure 3(A). The RNA secondary structure formed by the mutant (-134.50 kcal/mol) has a less negative minimum free energy value compared to the wild type (-136.30 kcal/mol), which makes it a less stable structure. While for the circular plots in Figure 3(B), C26735U mutation decreases the base pairing probabilities in flanking regions, which again predicted that the mutation decreases the folding stability of the RNA secondary structure.

In short, both C913U and C26735U mutations cause a more drastic change in RNA secondary structure. Given the shortcomings of prediction tools, it is necessary to check if the changes in RNA secondary structure affect the pathogenicity of SARS-CoV-2 using experimental approaches. Besides that, these two mutations also reduce the folding stability of the RNA secondary structure, which then affects the polypeptide translation and folding. There is evidence suggesting that stable RNA structures play a key role in reducing the translation speed to prevent “ribosomal traffic jams” so that the newly translated polypeptides can fold properly ^31^. Hence, both C913U and C26735U mutations increase the translation speed of SARS-CoV-2 RNA but they might cause the nascent polypeptide folding more prone to error during translation.

### RSCU analysis of SARS-CoV-2

Codon usage bias (CUB), which is non-random usage of synonymous codons, is common in all species. It is a phenomenon where some codons are preferred over others for a specific amino acid. SARS-CoV-2 replicates using host cell’s machinery and synthesizes its protein by utilizing host cellular components. Hence, codon usage bias may affect the replication of viruses ^32^.

Relative synonymous codon usage (RSCU) is a widely used statistical approach ^33^ that can be used to measure codon usage bias in coding sequences. The RSCU values of SARS-CoV-2 are shown in Table 2 and the most preferred codons for each amino acid are marked in bold. Stop codons (UAA, UAG, UGA) and codons which code for an amino acid uniquely (AUG, UGG) are excluded from RSCU analysis.

**Table 2.** RSCU values of SARS-CoV-2 genome.

Amino Acid	Synonymous Codons	RSCU
Ala	GCA	1.09
	GCC	0.58
	GCG	0.16
	**GCU**	**2.17**
Arg	**AGA**	**2.67**
	AGG	0.81
	CGA	0.29
	CGC	0.58
	CGG	0.19
	CGU	1.46
Asn	AAC	0.65
	**AAU**	**1.35**
Asp	GAC	0.72
	**GAU**	**1.28**
Cys	UGC	0.45
	**UGU**	**1.55**
Gln	**CAA**	**1.39**
	CAG	0.61
Glu	**GAA**	**1.44**
	GAG	0.56
Gly	GGA	0.82
	GGC	0.71
	GGG	0.12
	**GGU**	**2.34**
His	CAC	0.61
	**CAU**	**1.39**
Ile	AUA	0.92
	AUC	0.56
	**AUU**	**1.53**
Leu	CUA	0.66
	CUC	0.59
	CUG	0.30
	**CUU**	**1.75**
	UUA	1.63
	UUG	1.06
Lys	**AAA**	**1.31**
	AAG	0.69
Phe	UUC	0.59
	**UUU**	**1.41**
Pro	CCA	1.59
	CCC	0.29
	CCG	0.17
	**CCU**	**1.94**
Ser	AGC	0.36
	AGU	1.43
	UCA	1.67
	UCC	0.46
	UCG	0.11
	**UCU**	**1.96**
Thr	ACA	1.64
	ACC	0.38
	ACG	0.20
	**ACU**	**1.78**
Tyr	UAC	0.78
	**UAU**	**1.22**
Val	GUA	0.91
	GUC	0.56
	GUG	0.58
	**GUU**	**1.95**

Based on the RSCU values, the synonymous codons can be classified into five groups: i) codons with RSCU value equals to 1.0 are unbiased codons; ii) codons with RSCU value > 1.0 are codons preferred in a genome; iii) codons with RSCU value < 1.0 are codons less preferred in a genome; iv) codons with RSCU value > 1.6 are codons which are over-represented in a genome; v) codons with RSCU value < 1.6 are codons which are under-represented in a genome ^32^. There are 15 preferred codons (RSCU value > 1.0) and 11 over-represented codons (RSCU value > 1.6) in SARS-CoV-2 genome as shown in Table 2. The preferred codons in SARS-CoV-2 genome are GCA (Ala), CGU (Arg), AAU (Asn), GAU (Asp), UGU (Cys), CAA (Gln), GAA (Glu), CAU (His), AUU (Ile), UUG (Leu), AAA (Lys), UUU (Phe), CCA (Pro), AGU (Ser) and UAU (Tyr) while the over-represented codons are GCU (Ala), AGA (Arg), GGU (Gly), CUU (Leu), UUA (Leu), CCU (Pro), UCA (Ser), UCU (Ser), ACA (Thr), ACU (Thr), and GUU (Val). The presence of the preferred and over-presented codons in a genome increases the protein synthesis rate.

Table 3 shows the RSCU analysis of the top 10 synonymous mutations. The codons in bold in the ‘codon change’ column are the codons with higher RSCU value, which means they are more preferred in SARS-CoV-2 genome. Most of the mutations change the codon to a more preferred codon as shown in Table 3. Since it is presumed that preferred codons have a higher translation rate compared to nonpreferred codons ^34^, it is possible that most of the mutations may increase the translation efficiency of SARS-CoV-2, which may affect virus replication, transmission, and evolution.

**Table 3.** RSCU analysis of the top 10 synonymous mutations of SARS-CoV-2 genome.

ORF		Mutation	Codon Change
1a	nsp1	C313U	CUC -> **CUU**
	nsp2	C913U	UCC -> **UCU**
	nsp3	C3037U	UUC -> **UUU**
		C5986U	UUC -> **UUU**
	nsp4	C9286U	AAC -> **AAU**
1b	nsp12	C14676U	CCC -> **CCU**
		C15279U	CAC -> **CAU**
		U16176C	**ACU** -> ACC
	nsp14	C18877U	CUA -> **UUA**
M		C26735U	UAC -> **UAU**

## Conclusions

The effects of SARS-CoV-2 synonymous mutations in various aspects such as RNA folding and RNA stability of the virus were studied, even though they do not cause changes in amino acid residue of the protein. Most of the synonymous mutations identified in the SARS-CoV-2 genome are found to have a minor effect on RNA folding and RNA stability of the virus except for C913U and C26735U mutations. Due to the shortcomings of prediction tools, experimental studies are needed to give a more comprehensive understanding of the biological consequences of synonymous mutations on SARS-CoV-2 virus.

## Ethics and dissemination

No ethical approval is required for data analysis in this study (EA2702021).

## Data and software availability

### Underlying data

SARS-CoV-2 virus genome sequence data were obtained from the GISAID Database. The multiple alignment data can be assessed through FigShare.

 Figshare: MSA (SARS-CoV-2). https://doi.org/10.6084/m9.figshare.16681900.v1 ^35^

This project contains the following underlying data.

•   MSA_0.fasta (multiple sequence alignment of SARS-CoV-2 sequences obtained between 31-12-2019 and 31-05-2020.)

•   MSA_1.fasta (multiple sequence alignment of SARS-CoV-2 sequences obtained between 01-06-2020 and 15-10-2020.)

•   MSA_2.fasta (multiple sequence alignment of SARS-CoV-2 sequences obtained between 16-10-2020 and 31-01-2021.)

•   MSA_3.fasta (multiple sequence alignment of SARS-CoV-2 sequences obtained between 01-02-2021 to 22-03-2021.)

Data are available under the terms of the Creative Commons Attribution 4.0 International license (CC-BY 4.0).

### Software

The python code for the identification of SARS-CoV-2 genome mutations can be assessed through GitHub.
