# Prediction of the effects of the top 10 synonymous mutations from 26645 SARS-CoV-2 genomes of early pandemic phase

**DOI:** 10.12688/f1000research.72896.4

**Published:** 2024-09-18

**Authors:** Wan Xin Boon, Boon Zhan Sia, Chong Han Ng

**Affiliations:** 1 Faculty of Information Science and Technology, Multimedia University, Bukit Beruang, Melaka, 75450, Malaysia

**Keywords:** COVID-19, SARS-CoV-2, Synonymous mutations, RNA secondary structure

## Abstract

**Background:**

The emergence of severe acute respiratory syndrome coronavirus 2 (SARS-CoV-2) had led to a global pandemic since December 2019. SARS-CoV-2 is a single-stranded RNA virus, which mutates at a higher rate. Multiple works had been done to study nonsynonymous mutations, which change protein sequences. However, there is little study on the effects of SARS-CoV-2 synonymous mutations, which may affect viral fitness. This study aims to predict the effect of synonymous mutations on the SARS-CoV-2 genome.

**Methods:**

A total of 26645 SARS-CoV-2 genomic sequences retrieved from Global Initiative on Sharing all Influenza Data (GISAID) database were aligned using MAFFT. Then, the mutations and their respective frequency were identified. Multiple RNA secondary structures prediction tools, namely RNAfold, IPknot++ and MXfold2 were applied to predict the effect of the mutations on RNA secondary structure and their base pair probabilities was estimated using MutaRNA. Relative synonymous codon usage (RSCU) analysis was also performed to measure the codon usage bias (CUB) of SARS-CoV-2.

**Results:**

A total of 150 synonymous mutations were identified. The synonymous mutation identified with the highest frequency is C3037U mutation in the nsp3 of ORF1a. Of these top 10 highest frequency synonymous mutations, C913U, C3037U, U16176C and C18877U mutants show pronounced changes between wild type and mutant in all 3 RNA secondary structure prediction tools, suggesting these mutations may have some biological impact on viral fitness. These four mutations show changes in base pair probabilities. All mutations except U16176C change the codon to a more preferred codon, which may result in higher translation efficiency.

**Conclusion:**

Synonymous mutations in SARS-CoV-2 genome may affect RNA secondary structure, changing base pair probabilities and possibly resulting in a higher translation rate. However, lab experiments are required to validate the results obtained from prediction analysis.

## Introduction

In December 2019, coronavirus disease 2019 (COVID-19) cases first emerged from Wuhan, China ^1^. Soon after, rapid spread of COVID-19 has resulted in a serious global outbreak. COVID-19 is an infectious and potentially lethal disease caused by a newly found coronavirus strain, known as severe acute respiratory syndrome coronavirus 2 (SARS-CoV-2). The virus causes clinical manifestation ranging from asymptomatic to severe pneumonia and in the worst scenario, death ^2^. SARS-CoV-2 seems to have a higher transmission rate ^3^ but lower mortality rate ^2^ in comparison to Middle East respiratory coronavirus (MERS-CoV) and severe acute respiratory syndrome coronavirus (SARS-CoV).

SARS-CoV-2 is a single-stranded RNA virus with a genome size of 29,903 bases. In general, RNA viruses have a higher mutation rate than DNA viruses and this allows them to evolve rapidly, escaping the host immune defence response ^4^. Different SARS-CoV-2 variants with multiple synonymous and nonsynonymous mutations have been reported since the beginning of the outbreak ^5^. Some variants are classified as variants of concern (VOCs) since they are associated with the change in viral pathogenicity such as, higher disease severity, higher transmission rate, lower immunity response in the host as a consequence of the mutations ^6^. However, it is expected that most of these mutations in SARS-CoV-2 genome are either neutral or mildly deleterious ^7^. Numerous studies have been carried out to understand the molecular mechanisms of these nonsynonymous mutations on the functions of different SARS-CoV-2 proteins ^6^. For example, the Alpha variant (B.1.1.7) of SARS-CoV-2, first identified in the UK in late 2020, is characterized by several mutations, including the D614G mutation in the spike protein, which enhances its binding affinity to the ACE2 receptor ^8^. This variant exhibits increased transmissibility compared to the original virus, which led to its rapid spread globally ^9^. However, there are only a few studies on the synonymous mutations of SARS-CoV-2 genome ^10, 11^.

Synonymous mutations are also known as silent mutations because the nucleotide mutations result in a change in the RNA sequence without altering the amino acid sequence ^12^. Synonymous mutations have been suggested to have no functional consequence on the fitness of organisms and their evolution in long term ^13^. However, numerous recent studies had showed that synonymous mutations may affect the folding and stability of RNA structures ^14^. Interestingly a large scale study of synonymous mutations in multiple yeast genes has shown that most of synonymous mutations are not neutral, affecting the fitness of the cell ^15^. For RNA viruses, even though synonymous mutations generally do not change their pathogenicity directly, some studies reveal that synonymous mutations may affect the RNA secondary structure of the virus ^16^ and also change the codon usage bias of the genes in the virus ^17, 18^. The use of mRNA-based COVID-19 vaccines reduce the severity of the disease. However, mRNA molecule is susceptible to the degradation due to the presence of 2’ OH group in the ribose. To improve the stability of mRNA vaccine, Zhang *et al.* (2023) designed a novel algorithm, which optimizes the codon usage and RNA secondary structure by using synonymous codon ^19^.

The synonymous mutations play some important biological roles, which may affect viral fitness and pathogenicity. However, the study of biological consequences of synonymous mutations have been largely overlooked. In this study, we identified synonymous mutations of SARS-CoV-2 genome from early pandemic phase. We predicted the effects of these synonymous mutations of the top 10 highest frequency on RNA secondary structures and codon usage bias of SARS-CoV-2 genome. These findings allow the researchers to prioritize these mutations for function analysis in the future.

## Methods

### Sequence retrieval

30,229 SARS-CoV-2 genomic sequences were downloaded from GISAID database (Global Initiative on Sharing All Influenza Data, RRID:SCR_018251) ^20^ ranging from 31 December 2019 to 22 March 2021. SARS-CoV-2 genomic sequences were filtered by setting parameters to keep only sequences with complete genome and high coverage. The sequences were further filtered to remove those sequences with higher than 0.1% “N” unresolved nucleotides and ambiguous letters. A total of 3,584 sequences were removed by applying this filter. The reference sequence of SARS-CoV-2 genome ( NC_045512.2) ^21^ was retrieved in fasta format from NCBI database (NCBI, RRID:SCR_006472). It is a Wuhan isolate with a complete genome which comprises of 29,903 bases.

### Multiple sequence alignment

The rapid calculation available in MAFFT online server (MAFFT, version 7.467, RRID:SCR_011811) ^22^ was used to perform multiple sequence alignment (MSA) for 26,645 SARS-CoV-2 genomes. This option supports the alignment of more than 20,000 sequences with approximately 30,000 sites. The alignment length was kept, which means the insertions at the mutated sequences were removed, to keep the alignment length the same as the reference sequence. While other parameters were left as default.

### Identification of mutations and their frequency in SARS-CoV-2 genomes

A simple Python script was written to identify the mutations in 26,645 SARS-CoV-2 genomes. To determine whether the identified mutations are synonymous or nonsynonymous, MEGA X software, version 10.2.5 build 10210330 ( MEGA Software, RRID:SCR_000667) ^23^ was utilized to perform the translation for inspection purposes. The presence of amino acid changes was identified by referring to the genomic position of the nucleotide mutations. Synonymous mutations with the top 10 highest frequencies were generated.

### SARS-CoV-2 RNA secondary structure prediction

The RNA secondary structure of wild type and mutant sequences were predicted using RNAfold program, version 2.4.18 (Vienna RNA, RRID:SCR_008550) ^24^ with the incorporation of SHAPE reactivity data obtained from the study done by Manfredonia *et al.* (2020) ^25^. The RNA secondary structure prediction was performed using a sequence length of 250 nucleotides upstream and downstream of the mutation site. Other than RNAfold, another two programs which are IPknot++ version 2.2.1 (SCR_022557) ^26^, and MXFold2 (SCR_022558) ^27^ were also used to perform the RNA secondary structure prediction of SARS-CoV-2 wild type and mutants.

### Base pair probability estimation

To predict how the mutations affect RNA local folding, base pair probability was estimated by utilizing MutaRNA, version 1.3.0 (MutaRNA, RRID:SCR_021723) ^28^. MutaRNA is a web-based tool that allows prediction and visualization of the structure changes induced by a single nucleotide polymorphism (SNP) in an RNA sequence. It includes the base pair probabilities within RNA molecule of both wild type and mutant. The parameters used in MutaRNA were set as default except the window size was changed to 501nt.

### Relative Synonymous Codon Usage (RSCU)

Relative synonymous codon usage (RSCU) represents the ratio of the observed frequency of codons appearing in a gene to the expected frequency under equal codon usage. RSCU is calculated using the formula:

$$RSCU_{i}=\frac{X_{i}}{\frac{1}{n}\sum_{i=1}^{n}X_{i}},$$

where X _i_ implies the number of occurrences of codon i and n stands for the number of synonymous codons encoded for that particular amino acid.

## Results and discussion

A synonymous mutation is a change in the nucleotide that does not cause any changes in the encoded amino acid. Synonymous mutations were previously considered to be less important, but they are now proven to have some effects on RNA folding, RNA stability, miRNA binding and translational efficiency ^29^. Synonymous mutations may have significant effects on the adaptation, virulence, and evolution of RNA viruses ^30^. Another study done also indicated that synonymous mutations have association with more than 50 human diseases such as hemophilia B, tuberculosis (TB), cystic fibrosis (CF), Alzheimer, schizophrenia, chronic hepatitis C and so on ^31^. All these studies show that increasing importance has been associated with synonymous mutations over these years. Hence, it is necessary for us to study the effects of synonymous mutations of SARS-CoV-2 genome.

### Identification of SARS-CoV-2 synonymous mutations

**Figure 1.**
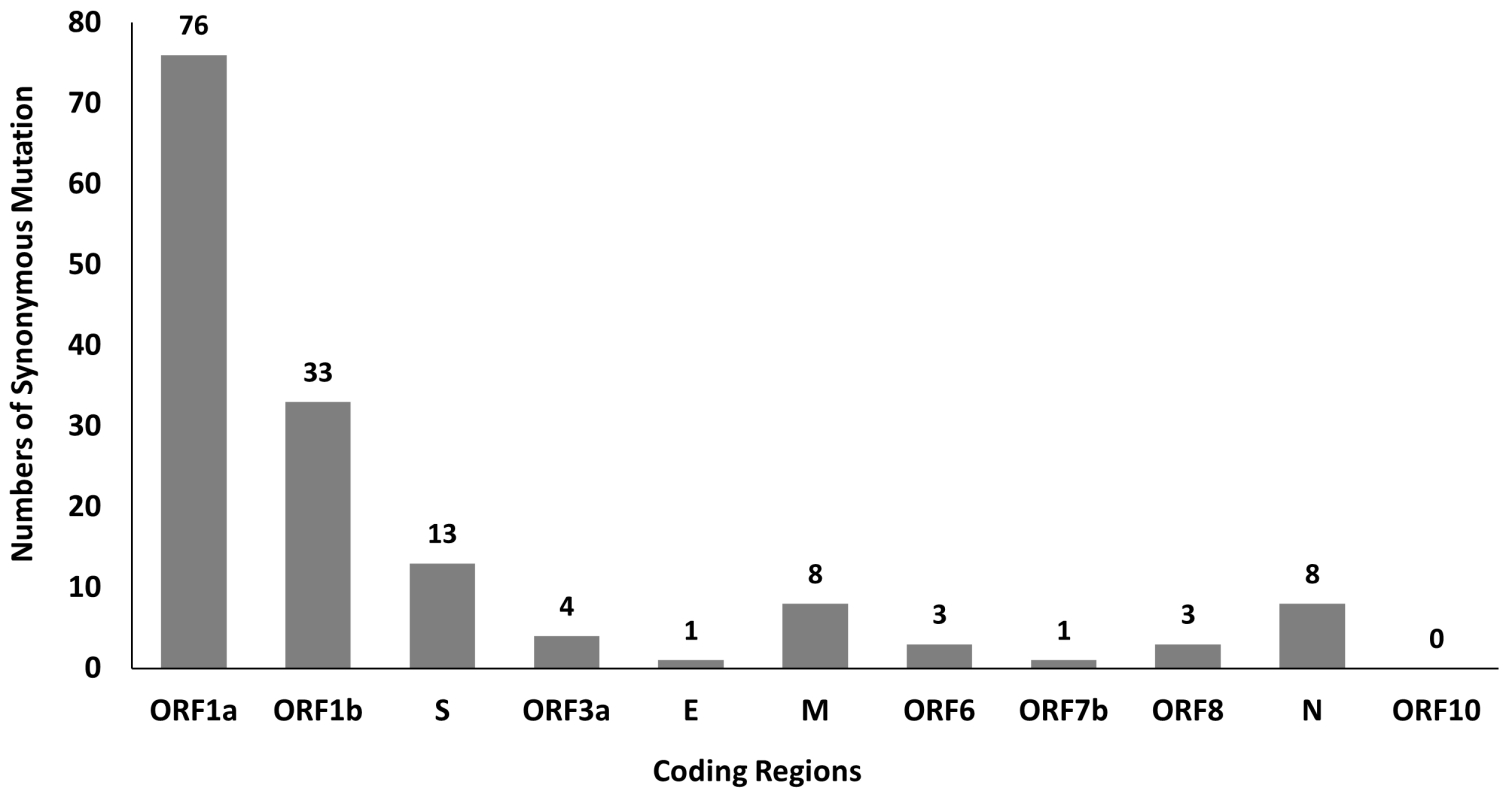
Distribution of SARS-CoV-2 synonymous mutations in 11 coding regions.

A total of 381 mutations were found in SARS-CoV-2 genomes by using python script, in which 150 of them are synonymous mutations. The distribution of these 150 synonymous mutations in 11 coding regions is shown in Figure 1. Among these mutations, ORF1a and ORF1b have a higher number of synonymous mutations at 76 and 33, respectively, which might be due to their longer sequence length. Besides that, our findings also show high C to U mutation rate in SARS-CoV-2 genome and this mutational skews are in line with multiple studies ^32– 35^. The high C to U mutation rate may be driven by host APOBEC-mediated RNA editing system and overexpression of APOBEC3 protein promotes viral replication and propagation in the human colon epithelial cell line ^36^. These mutational skews are necessary to be considered when deducing the selection acting on synonymous variants in SARS-CoV-2 evolution ^11^. Synonymous mutations are assumed subject to a lower selective pressure than nonsynonymous mutations, presumably the purifying selection force has stronger negative impact on the frequencies of nonsynonymous mutations. Interestingly there may be some selection force on synonymous mutations shown by a few studies, suggesting that these synonymous mutations are not random and neutral, may have some biological impact on viral fitness ^11, 32, 37^.

The synonymous mutations in SARS-CoV-2 genomes with the top 10 highest frequency obtained from the analysis of 150 synonymous mutations were listed in Table 1. Our sequence samples are obtained from December 2019 to March 2021 and this period overlapped with the peak of Alpha variant (B.1.1.7) outbreak ^5^. The defining synonymous SNPs of Alpha variant include C241T, C913T, C3037T, C5986T, C14676T, C15279T and T16176C ^5^, and all except C241T are reported in our study as well. As shown in Table 1, synonymous mutations with the highest frequency identified from SARS-CoV-2 genomes is C3037U mutation located in nsp3 of ORF1a, followed by C313U mutation in nsp1 of ORF1a and C9286U mutation in nsp4 of ORF1a. Mutations with higher frequency are mostly found in ORF1a and ORF1b. Although there are some overlapping ORFs in the SARS-CoV-2 genome, such as ORF1a and ORF1b, ORF3a and ORF3c ^38^, the top 10 highest frequency synonymous mutations are not located in these overlapping sites. It is of great interest to find out the effect of these top 10 synonymous mutations on SARS-CoV-2 genome. However, it is important to take note that the high frequency of some mutations is not necessarily due to their positive effects. They may emerge during early stage of pandemic and are transmitted to all of their descendants, even though they have no or little effect on viral fitness ^39^.

**Table 1.** SARS-CoV-2 synonymous mutations with the top 10 highest frequency.

ORF		Position	Nucleotide Variation	Frequency
1a	nsp1	313	C > U	8212
	nsp2	913	C > U	2820
	nsp3	3037	C > U	24651
		5986	C > U	2860
	nsp4	9286	C > U	3880
1b	nsp12	14676	C > U	2859
		15279	C > U	2842
		16176	U > C	2838
	nsp14	18877	C > U	2603
M		26735	C > U	2432

Similar to another companion paper, which focuses on the prediction analysis of nonsynonymous mutations of SARS-CoV-2 proteins ^40^, the same SARS-CoV-2 virus genome data from GISAID database ranging from 1st January 20 to 22 March 21 were used in this study. The data collection time was overlapping with the period when the frequency of alpha variant reached the highest numbers around March–May 21 ^34^. There are seven synonymous mutations identified as the defining mutations in the alpha variant, of which all except C241T are also reported in our study. Due to the rapid evolution of SARS-CoV-2 genome, it is beyond the scope of our study to keep track SARS-CoV-2 mutational profile and to predict the consequences of these mutations. Two independent studies reported that alpha or alpha-like SARS-CoV-2 variants are circulating among wild deer population in North America in late 2021 ^41, 42^. Although there is no reported case of viral spillback from deer to human transmission, we can’t simply rule out this possibility yet. Hence, our findings remain relevant despite of not using the latest genome dataset.

### RNA secondary structure prediction and base pair probability estimation analysis

SARS-CoV-2 virus can form highly structured RNA elements, which may affect viral replication, discontinuous transcription and translation ^43, 44^. For example, SARS-CoV-2 forms a three-stemmed pseudoknot structure to promote programmed -1 ribosomal frameshifting to increase the synthesis of the proteins required for viral replication ^43, 44^. There are numerous high throughput studies on the characterization of RNA secondary structure of SARS-CoV-2 genome ^25, 45– 48^. In these recent high throughput studies, the RNA secondary structures of SARS-CoV-2 genome were determined experimentally using chemical probing methods, such as SHAPE-MaP ^25, 45^ or proximity ligation methods, such as RIC-seq ^47^, COMRADES ^48^. Although these data are very useful to determine the RNA secondary structures of SARS-CoV-2 virus, there is very little study on the effect of the synonymous mutations on RNA secondary structure, which may be beneficial or deleterious to the viral fitness. Therefore, we performed RNA secondary structure prediction and base pair probability estimation analysis of these top 10 highest frequency of synonymous mutations.

To improve the outcome of the study, multiple RNA secondary structure prediction tools, namely RNAfold with SHAPE reactivity data ^24^, IPknot++ ^26^ and MXfold2 ^27^ were applied in our study. In addition, MutaRNA analysis tool was used to estimate the base pair potential of the wild type and mutant sequences. RNAfold with SHAPE reactivity data uses thermodynamic approach to calculate the minimum free energy for the most probable RNA secondary structure by incorporating the nucleotide reactivity data derived from the experiments. If the reactivity value is high, the nucleotide is less likely to be paired, or vice versa. SARS-CoV-2 virus can form pseudoknot structures, which promote ribosomal frameshifting ^44^. However, many RNA secondary structure prediction programmes don’t predict pseudoknot structure since the calculation is computationally demanding. IPknot++ is one of the few programmes, which can predict pseudoknot structure. MXfold2 predicts RNA secondary structure using deep learning method with a large amount of training dataset.

Although different tools may produce similar results for identical RNA sequences, it's important to note that there can be variations in prediction outputs due to differences in algorithms, thermodynamic parameter settings, inclusion of pseudoknot calculation, incorporation of experimental data and the assumptions of each tool. RNAfold, IPknot++ and MXfold2 apply the nearest neighbour model, using different thermodynamic parameters. In addition, MXfold2 implements deep learning models with max margin framework ^49^. Multiple experimental genome-wide mapping of RNA secondary structures studies showed that 5’ UTR of SARS-CoV-2 RNA genome forms 7 conserved stem-loop structures, and in some studies, 8 SLs, depending on the sequence length ^25, 45– 48, 50^. To demonstrate the usability of prediction tools, we predicted RNA secondary structure of the sequence of 5’ UTR (1-480 nt) of SARS-CoV-2 (Extended data 1) ^51^. The RNA secondary structures predicted by RNAfold with SHAPE data, IPknot++ and MXfold2 are similar, especially SL1, SL5-8 regions and they are comparable to most of the published experimental data ^25, 45– 48, 50^. Both RNAfold with SHAPE data, and MXfold2 successfully predicted SL4, but IPknot++ predicted SL4 with pseudoknot structure, which has not been reported in other studies. Interestingly the result obtained from RNAfold without SHAPE data is quite different, possibly due to the missing experimental data. In addition, it has been shown that SARS-CoV-2 may adopt different RNA secondary structure conformations ^7, 19, 36, 37, 39, 41^. Our study is aimed to predict if the sSNP may affect RNA secondary structure and the outcomes allow us to prioritize variants for the experiment functional studies in the future. Using multiple prediction tools may help to increase the accuracy and reliability of the prediction result. The prediction results for all 10 synonymous mutations using these 3 tools and the base pair probability estimation results are summarized in the Table 2 (✓ - changes, × - no change). The results for all 10 synonymous mutations predicted with RNAfold, IPknot++ and MXfold2 are available in Extended data 2, 3 and 4, respectively ^51^. The base pair probabilities for all 10 synonymous mutations are shown as circular plots in Extended data 5 ^51^. The darker the edge is, the more likely the two connected bases to form base pair. Of these 10 synonymous mutations, four mutants which are all located in ORF1ab, namely C913U, C3037U, U16176C and C18877U mutants show pronounced changes between wild types and mutants in all 3 prediction tools, suggesting these synonymous mutations may have some biological impact on viral fitness. Having say that, it is also possible that other mutants with only one or two changes predicted by these analyses, may also affect RNA secondary structures, having some impact on viral fitness. It has been shown that SARS-CoV-2 virus can form elaborated RNA secondary structures at 5’ and 3’UTRs, and frameshifting element (FSE), located between the boundary of ORF1a and ORF1ab ^7, 19, 36, 37, 39, 41^. The 5’ UTR of SARS-CoV-2 is important for viral mRNA stability ^52^ and protein translation ^53^ while the 3’ UTR may be involved in viral proliferation in the host cell ^54^. Interestingly it has been observed that base substitution type, transitions from C to U base occurred at higher frequency in the stem region of RNA secondary structure of 5’ and 3’ UTR of SARS-CoV-2 genome, possibly due to the less detrimental effect on the structure ^34^. The FSE can form pseudoknot structures, which regulate the relative protein expression of ORF1a and ORF1ab during viral infection ^43, 44^.

**Table 2.** Summary of RNA secondary structure prediction and base pair probability estimation analysis of SARS-CoV-2 synonymous mutations.

	RNAfold (SHAPE)	IPknot++	MXFold2	MutaRNA
C313U	**×**	**×**	**×**	**×**
C913U	**✓**	**✓**	**✓**	**✓**
C3037U	**✓**	**✓**	**✓**	**✓**
C5986U	**✓**	**×**	**✓**	**✓**
C9286U	**×**	**✓**	**×**	**✓**
C14676U	**✓**	**✓**	**×**	**✓**
C15279U	**×**	**✓**	**×**	**×**
U16176C	**✓**	**✓**	**✓**	**✓**
C18877U	**✓**	**✓**	**✓**	**✓**
C26735U	**✓**	**✓**	**×**	**✓**

**Figure 2.**
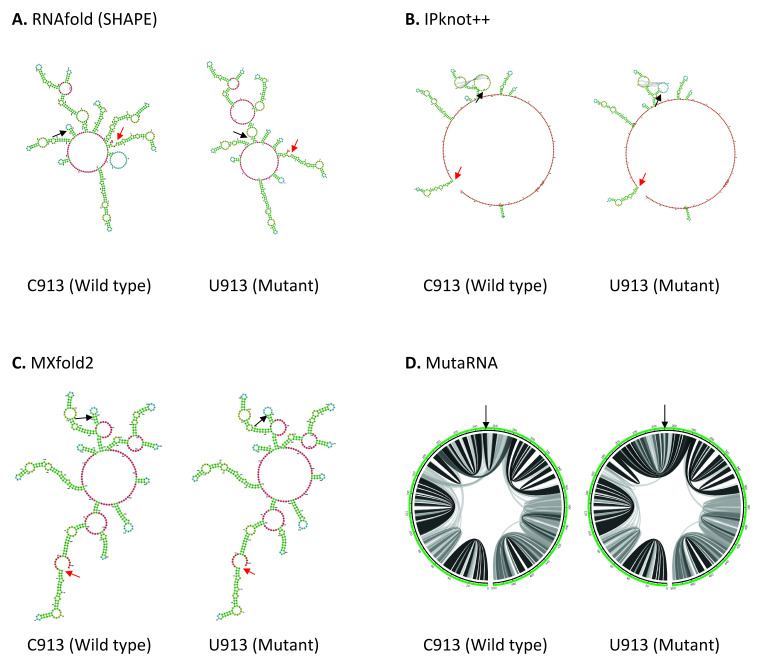
The effect of C913U mutation on RNA secondary structure of nsp2 in ORF1a. ( **A**) RNA secondary structure of C913 wild type and U913 mutant predicted using RNAfold. ( **B**) RNA secondary structure of C913 wild type and U913 mutant predicted using IPknot++. ( **C**) RNA secondary structure of C913 wild type and U913 mutant predicted using MXfold2. ( **D**) MutaRNA circular plots of base pairing probabilities of C913 wild type and U913 mutant. The black arrow indicates the position of WT and mutated nucleotides while the red arrow indicates the starting position of the query sequence.

Other than 5’ and 3’ UTRs, Huston *et al.* (2021) found that ORF1ab region forms extensive RNA secondary structure network ^45^. Coincidentally all four mutations, C913U, C3037U, U16176C and C18877U reported in our study are located within ORF1ab. C913U mutation is found in the Nsp2, near the start codon (position 806) in ORF1a in SARS-CoV-2 genome. As shown in Figure 2, the wild type structures predicted by RNAfold and MXfold2 shares some degree of similarity around position 95–330 of 501 base long structure. C913U mutation has a pronounced effect on RNA secondary structure predicted by RNAfold. C913U mutation results in the appearance or disappearance of multiple loops, not only at the nearby mutated residue, but also at the sites further apart, suggesting this mutation may affect its long-range RNA interaction. While MXfold2 predicts that U913 mutant forms a shorter stem and a larger hairpin loop compared to C913 wild type. However, the structure predicted by IPknot++ is quite different from others, in which, C913U results in change of pseudoknot structure. Figure 2D shows that the base pair interactions of wild type RNA are changed substantially by U913 mutation. Previously it has been shown that Nsp2 protein suppresses host immune response by inhibiting the mRNA translation of interferon gene ^55^. Although C913U mutation does not alter the amino acid residue of Nsp2 protein, it may be worthwhile to see if this C913U mutation plays a direct or indirect role in host immune response through Nsp2 protein. Since C913U is near to Nsp1 and Nsp2 protein boundaries, the altered RNA secondary structure may affect ribosome stalling, which, in turn, affect folding of nascent polypeptides and translation initiation. In addition, Nsp1 protein facilitates viral propagation by inhibiting host protein translation machinery ^56^ and promoting host mRNA degradation ^57^. It will be interesting to investigate if the C913U mutation affects these functions. 

**Figure 3.**
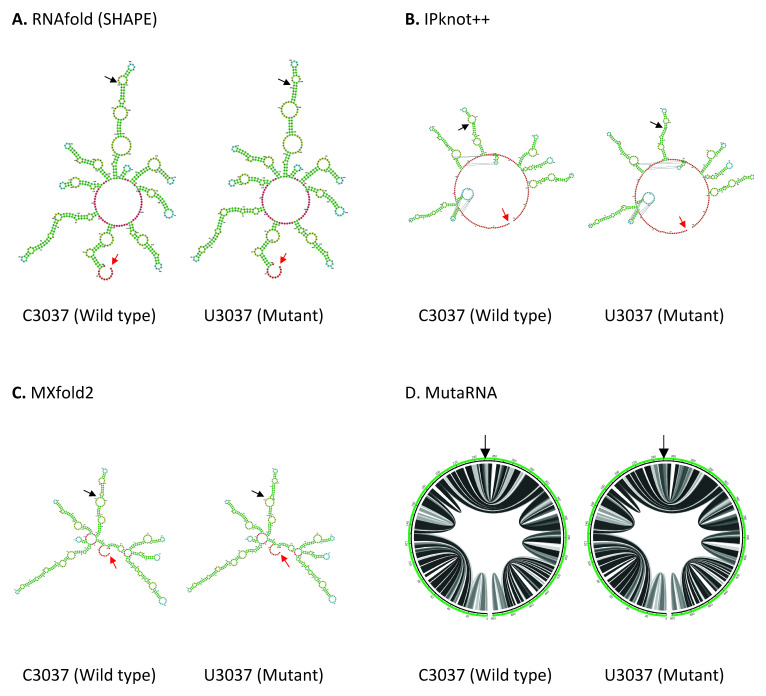
The effect of C3037U mutation on RNA secondary structure of nsp3 in ORF1a. ( **A**) RNA secondary structure of C3037 wild type and U3037 mutant predicted using RNAfold. ( **B**) RNA secondary structure of C3037 wild type and U3037 mutant predicted using IPknot++. ( **C**) RNA secondary structure of C3037 wild type and U3037 mutant predicted using MXfold2. ( **D**) MutaRNA circular plots of the base pairing probabilities of C3037 wild type and U3037 mutant. The black arrow indicates the position of WT and mutated nucleotides while the red arrow indicates the starting position of the query sequence.

C3037U mutation is found in the Nsp3 in ORF1a. As shown in Figure 3, both IPknot++ and MXfold2 predict that U3037 mutant forms longer stem and smaller internal loop compared to wild type. On the contrary, RNAfold predicts that a small internal loop fuses into a bigger internal loop in U3037 mutant. MutaRNA circular plot shows that there is some minor difference in base pair probabilities between C3037 wild type and U3037 mutant. Nsp3 is a papain-like protease, which hydrolyzes several Nsp proteins, involved in viral replication ^58^. Hence, we should investigate the effect of this mutation on its cleavage activity, probably through the change in transcription or translation level of Nsp3.

**Figure 4.**
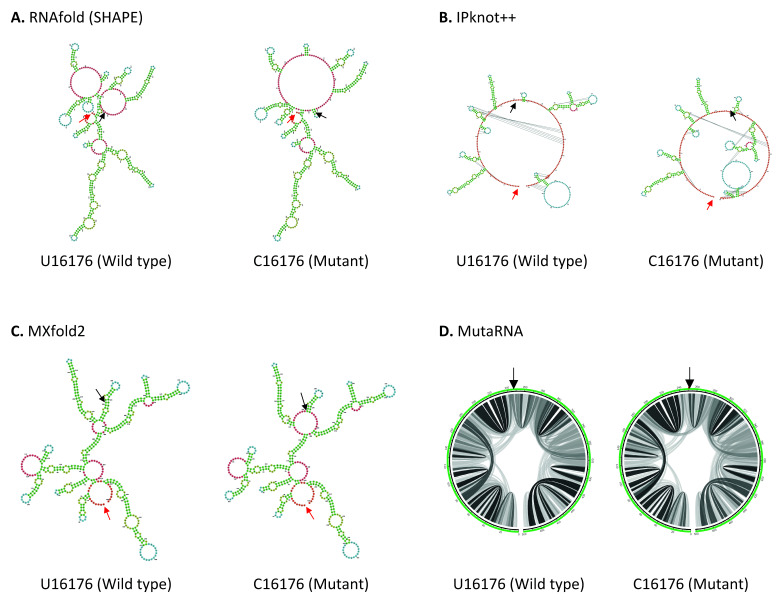
The effect of U16176C mutation on RNA secondary structure of nsp12 in ORF1b. ( **A**) RNA secondary structure of U16176 wild type and C16176 mutant predicted using RNAfold. ( **B**) RNA secondary structure of U16176 wild type and C16176 mutant predicted using IPknot++. ( **C**) RNA secondary structure of U16176 wild type and C16176 mutant predicted using MXfold2. ( **D**) MutaRNA circular plots of the base pairing probabilities of U16176 wild type and C16176 mutant. The black arrow indicates the position of WT and mutated nucleotides while the red arrow indicates the starting position of the query sequence.

U16176C mutation is located in the Nsp12, close to the boundary of Nsp12 and Nsp13 genes in ORF1b. As shown in Figure 4, U16176C mutation results in a drastic change in RNA secondary structure predicted using RNAfold. IPknot++ predicts C16176 mutant forms new pseudoknot structures, which are absent in wild type U16176. On the other hand, MXfold2 predicts C16176 mutant forms a larger multi-branched loop and a shorter stem compared to wild type. Similarly, MutaRNA result shows C16176 mutant affects base pair potential at multiple sites. Nsp12 is one of the subunits of RNA-dependent RNA polymerase (RdRp), which is required for RNA synthesis ^59^. A study showed that a 1.4-kb-long SARS-CoV-2 RNA sequence (residues 15071–16451) located in the Nsp12 and Nsp13 regions is required to facilitate viral RNA packaging ^60^. Since U16176C mutation may affect RNA secondary structure, it will be interesting to see if it affects viral RNA packaging. U16176C together with C14676U and C15279U have very similar number of frequencies as shown in Table 1. Interestingly IPknot++ predicted all of them result in changes in pseudoknot structure as shown in Extended data 3. We speculated that these three sSNPs may be functionally related. These mutations are located downstream of the frameshifting element (residues 13405–13488) and this element forms a pseudoknot to promote ribosomal frameshifting during viral replication ^61^. It has been demonstrated that synonymous mutations affect both RNA secondary structure of the ribosomal frameshift signal and frameshifting efficiency in SARS-CoV virus ^62^. Another study had shown that this ribosomal frameshifting structure in SARS-CoV-2 virus involves long-range sequence interaction of 1.5 kb ^48^. It remains to be seen whether the long-range sequence interaction for ribosomal frameshifting can go beyond 1.5kb long.

**Figure 5.**
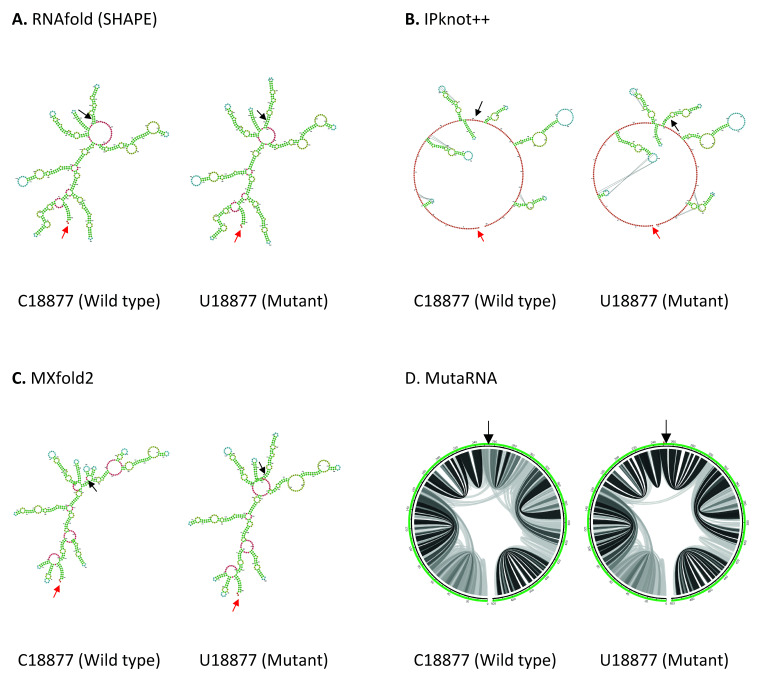
The effect of C18877U mutation on RNA secondary structure of nsp14 in ORF1b. ( **A**) RNA secondary structure of C18877 wild type and U18877 mutant predicted using RNAfold. ( **B**) RNA secondary structure of C18877 wild type and U18877 mutant predicted using IPknot++. ( **C**) RNA secondary structure of C18877 wild type and U18877 mutant predicted using MXfold2. ( **D**) MutaRNA circular plots of the base pairing probabilities of C18877 wild type and U18877 mutant. The black arrow indicates the position of WT and mutated nucleotides while the red arrow indicates the starting position of the query sequence.

C18877U mutation is located in Nsp14 in ORF1b. As shown in Figure 5, an additional internal loop is formed in U18877 mutant predicted by RNAfold. IPknot++ predicts U18877 mutant forms extra internal loops and longer hairpin near the mutated residue and it also affects the pseudoknot structure at 2 different sites further from the mutated residue. While MXfold2 predicts U18877 mutant forms one hairpin with multiple loops instead of one hairpin as seen in wild type. The changes at multiple base pairing sites due to the U18877 mutation is also observed in MutaRNA circular plot. Nsp14 is important to maintain high fidelity during viral RNA synthesis ^63^.

### RSCU analysis of SARS-CoV-2

Other than affecting RNA secondary structure, it has been shown that synonymous mutations may affect protein translation efficiency and accuracy through the formation of codon usage bias (CUB), which is non-random usage of synonymous codons, common in all species ^64^. It is a phenomenon where some codons are preferred over others for a specific amino acid. SARS-CoV-2 replicates using host cell’s machinery and synthesizes its protein by utilizing host cellular components. Hence, codon usage bias may affect the replication of viruses ^65^.

Relative synonymous codon usage (RSCU) is a widely used statistical approach ^66^ that can be used to measure codon usage bias in coding sequences. The RSCU values of SARS-CoV-2 are shown in Table 3 and the most preferred codons for each amino acid are marked in bold. Stop codons (UAA, UAG, UGA) and codons which code for an amino acid uniquely (AUG, UGG) are excluded from RSCU analysis.

**Table 3.** RSCU values of SARS-CoV-2 genome.

Amino Acid	Synonymous Codons	RSCU
Ala	GCA	1.09
	GCC	0.58
	GCG	0.16
	**GCU**	**2.17**
Arg	**AGA**	**2.67**
	AGG	0.81
	CGA	0.29
	CGC	0.58
	CGG	0.19
	CGU	1.46
Asn	AAC	0.65
	**AAU**	**1.35**
Asp	GAC	0.72
	**GAU**	**1.28**
Cys	UGC	0.45
	**UGU**	**1.55**
Gln	**CAA**	**1.39**
	CAG	0.61
Glu	**GAA**	**1.44**
	GAG	0.56
Gly	GGA	0.82
	GGC	0.71
	GGG	0.12
	**GGU**	**2.34**
His	CAC	0.61
	**CAU**	**1.39**
Ile	AUA	0.92
	AUC	0.56
	**AUU**	**1.53**
Leu	CUA	0.66
	CUC	0.59
	CUG	0.30
	**CUU**	**1.75**
	UUA	1.63
	UUG	1.06
Lys	**AAA**	**1.31**
	AAG	0.69
Phe	UUC	0.59
	**UUU**	**1.41**
Pro	CCA	1.59
	CCC	0.29
	CCG	0.17
	**CCU**	**1.94**
Ser	AGC	0.36
	AGU	1.43
	UCA	1.67
	UCC	0.46
	UCG	0.11
	**UCU**	**1.96**
Thr	ACA	1.64
	ACC	0.38
	ACG	0.20
	**ACU**	**1.78**
Tyr	UAC	0.78
	**UAU**	**1.22**
Val	GUA	0.91
	GUC	0.56
	GUG	0.58
	**GUU**	**1.95**

Based on the RSCU values, the synonymous codons can be classified into five groups: i) codons with RSCU value equals to 1.0 are unbiased codons; ii) codons with RSCU value > 1.0 are codons preferred in a genome; iii) codons with RSCU value < 1.0 are codons less preferred in a genome; iv) codons with RSCU value > 1.6 are codons which are over-represented in a genome; v) codons with RSCU value < 1.6 are codons which are under-represented in a genome ^65^. There are 15 preferred codons (RSCU value > 1.0) and 11 over-represented codons (RSCU value > 1.6) in SARS-CoV-2 genome as shown in Table 3. The preferred codons in SARS-CoV-2 genome are GCA (Ala), CGU (Arg), AAU (Asn), GAU (Asp), UGU (Cys), CAA (Gln), GAA (Glu), CAU (His), AUU (Ile), UUG (Leu), AAA (Lys), UUU (Phe), CCA (Pro), AGU (Ser) and UAU (Tyr) while the over-represented codons are GCU (Ala), AGA (Arg), GGU (Gly), CUU (Leu), UUA (Leu), CCU (Pro), UCA (Ser), UCU (Ser), ACA (Thr), ACU (Thr), and GUU (Val). The presence of the preferred and over-presented codons in a genome increases the protein synthesis rate.

Table 4 shows the RSCU analysis of the top 10 synonymous mutations. The codons in bold in the ‘codon change’ column are the codons with higher RSCU value, which means they are more preferred in SARS-CoV-2 genome. Most of the mutations change the codon to a more preferred codon as shown in Table 4. Nine of the ten synonymous mutations involve changes from C to U nucleotides and eight of them are located at the third position of codon, suggesting these changes are not random and possibly subjected to some selection pressure. In agreement with our study, the excessive changes of C to U nucleotides in SARS-CoV-2 genome has been reported in multiple studies ^32– 35^. Since the preferred codons may have a better translation efficiency and accuracy compared to the nonpreferred codons ^64^, it is possible that most of these mutations may increase the viral fitness. While a study show that RNA secondary structures may be functionally linked to protein translation based on the evidence obtained from experimental work ^67^, it is difficult for us to establish the connection solely using *in silico* studies.

**Table 4.** RSCU analysis of the top 10 synonymous mutations of SARS-CoV-2 genome.

ORF		Mutation	Codon Change
1a	nsp1	C313U	CUC -> **CUU**
	nsp2	C913U	UCC -> **UCU**
	nsp3	C3037U	UUC -> **UUU**
		C5986U	UUC -> **UUU**
	nsp4	C9286U	AAC -> **AAU**
1b	nsp12	C14676U	CCC -> **CCU**
		C15279U	CAC -> **CAU**
		U16176C	**ACU** -> ACC
	nsp14	C18877U	CUA -> **UUA**
M		C26735U	UAC -> **UAU**

## Conclusions

The effects of SARS-CoV-2 synonymous mutations in various aspects such as RNA secondary structure and codon usage bias were studied, even though they do not cause changes in amino acid residue of the protein. C913U, C3037U, U16176C and C18877U mutants show pronounced changes between wild type and mutant predicted in all 3 RNA secondary structure prediction tools, suggesting these mutations may have some biological impact on viral fitness. In addition, these mutations showed changes in base pair potential estimated by MutaRNA. All mutations except U16176C change the codon to a more preferred codon, which may result in higher translation efficiency. Due to the shortcomings of prediction tools, experimental studies, such as protein translation assays, RNA packaging assays, are needed to give a more comprehensive understanding of the biological consequences of synonymous mutations on SARS-CoV-2 virus.

## Ethics and dissemination

No ethical approval is required for data analysis in this study (EA2702021).

## Data Availability

SARS-CoV-2 virus genome sequence data were obtained from the GISAID Database. The multiple alignment data can be assessed through FigShare. Figshare: MSA (SARS-CoV-2). https://doi.org/10.6084/m9.figshare.20486178.v1 ^68^ Figshare: **RNA secondary structure prediction and base pair probability estimation analysis** https://doi.org/10.6084/m9.figshare.20486166.v6 ^52^ Extended data 1. Comparation of RNA secondary structure of SARS-CoV-2 5’ UTR (1-480 nt) predicted using RNAfold without SHAPE data, RNAfold with SHAPE data, IPknot++, MXfold2 Extended data 2. The RNA secondary structure of SARS-CoV-2 genome predicted using RNAfold. Extended data 3. The RNA secondary structure of SARS-CoV-2 genome predicted using IPknot++. Extended data 4. The RNA secondary structure of SARS-CoV-2 genome predicted using MXfold2. Extended data 5. The base pair probabilities of SARS-CoV-2 genome estimated using MutaRNA Data are available under the terms of the Creative Commons Attribution 4.0 International license (CC-BY 4.0).

## Software

The python code for the identification of SARS-CoV-2 genome mutations can be assessed through GitHub.
