# Supplementary material for: Prediction of the effects of the top 10 synonymous mutations from 26645 SARS-CoV-2 genomes of early pandemic phase
Source: F1000Res. 2024 Sep 18;10:1053. Originally published 2021 Oct 18. [Version 4] doi: 10.12688/f1000research.72896.4 (PMC11391198; doi:10.12688/f1000research.72896.4)
Supplement: Peer review history

# Reviewer report: Reviewer response for version 4

**DOI:** 10.5256/f1000research.171513.r324668

**Published:** 2024-09-27

**Authors:** Diego Forni

**Affiliations:** 1 Scientific Institute IRCCS E Medea, Bosisio Parini, Italy

I would like to thank the authors for answering my questions.

 I still believe that it is not optimal to report counts without taking into account ORD length in figure 1.

 I still also believe that differences among RNA secondary structures are relevant and I am not really sure that "C913U, C3037U, U16176C and C18877U mutants show pronounced changes between wild type and mutant" as stated in the conclusion.

 Finally the two sentences starting with "Synonymous mutations are assumed subject to a lower selective pressure than nonsynonymous mutations, presumably.." in the introduction are not clear and need to be better explained.

Is the work clearly and accurately presented and does it cite the current literature?

Partly

If applicable, is the statistical analysis and its interpretation appropriate?

Yes

Are all the source data underlying the results available to ensure full reproducibility?

Yes

Is the study design appropriate and is the work technically sound?

Partly

Are the conclusions drawn adequately supported by the results?

Partly

Are sufficient details of methods and analysis provided to allow replication by others?

Partly

Reviewer Expertise:

viral genomics, viral evolution

I confirm that I have read this submission and believe that I have an appropriate level of expertise to confirm that it is of an acceptable scientific standard, however I have significant reservations, as outlined above.

# Reviewer report: Reviewer response for version 3

**DOI:** 10.5256/f1000research.162881.r304727

**Published:** 2024-09-11

**Authors:** Tamar Schlick

**Affiliations:** 1 Department of Chemistry and Courant Institute of Mathematical Sciences, New York University, New York, New York, USA

In this work, synonymous mutations of the SARS-CoV-2 genome are explored, with the rationale that these mutations impact function through altered RNA folding, despite unaltered protein products. Specifically, the researchers find 150 synonymous mutations after performing multiple sequence alignment and examining the protein products, and mapped the distributions in the coding regions. RNA secondary structure predictions and base pair probability calculations are then presented for 4 mutations C913U, C3037U, U16176C and C18877U that show pronounced changes between wildtype and mutant structures. Different prediction tools were used to confirm the structure predictions. The value of the study, while interesting, is limited because the long RNA lengths used make the disparate predicted structures unreliable. If the Information Classification: General authors examine some mutant systems experimentally with chemical reactivity like SHAPE or DMS, the results may be more meaningful.

Is the work clearly and accurately presented and does it cite the current literature?

Yes

If applicable, is the statistical analysis and its interpretation appropriate?

Partly

Are all the source data underlying the results available to ensure full reproducibility?

Yes

Is the study design appropriate and is the work technically sound?

Partly

Are the conclusions drawn adequately supported by the results?

Partly

Are sufficient details of methods and analysis provided to allow replication by others?

Yes

Reviewer Expertise:

NA

I confirm that I have read this submission and believe that I have an appropriate level of expertise to confirm that it is of an acceptable scientific standard, however I have significant reservations, as outlined above.

# Reviewer report: Reviewer response for version 3

**DOI:** 10.5256/f1000research.162881.r304726

**Published:** 2024-07-27

**Authors:** Diego Forni

**Affiliations:** 1 Scientific Institute IRCCS E Medea, Bosisio Parini, Italy

In this manuscript, Dr Boon et al. analyzed the most common synonymous mutations of  early pandemic SARS-CoV-2 genomes. They identified substitutions that influence the RNA secondary structure surrounding these positions. The authors have already modified and updated their manuscript based on previous comments, still there are few open questions in my opinion.

 First of all, it is clear that the dataset is not up to date and it will not be changed, still this should be clearly stated, starting from the title, reporting or the time period or something like "at the beginning of the pandemic''.

 - Following this, also the introduction section should be focused on what we know about genomic variability and viral evolution in the time period being analyzed herein. If I understood well, most of these sequences belong to the alpha variant.

 - Moreover, " variant of concern" (abbreviated VOC not VOI as stated in the text) refers to a specific lineage that carries several and distinct mutations. As it is stated in the text now it seems that a single mutation is a VOC. Please check it.

 - It is expected that most mutations are found in ORF1ab, as this covers most of the genome, so counts should be normalized by ORF length (e.g. fig.1).

 - Table 1 should report mutation frequency not count. 

 - Authors should discuss more why different predictors give very different results in terms of rna secondary structure, and how this can affect their analysis.

 - Why did the author select 250 bp flanking positions for their prediction? Which is the rationale? How does this influence the results?

 -  Are there any other mutations in the 501 nucleotide regions? Could mutations have an impact on predictions?

 - How did the authors handle overlapping/internal ORFs? In these situations a syn mutations could also be non-synonymous  for the other ORF. I do not think this is the case for the top 10 mutations, but still this should be explained.

 - The CUB section seems to me not really linked to the rest of the analyses and could be expanded more.

Is the work clearly and accurately presented and does it cite the current literature?

Partly

If applicable, is the statistical analysis and its interpretation appropriate?

Yes

Are all the source data underlying the results available to ensure full reproducibility?

Yes

Is the study design appropriate and is the work technically sound?

Partly

Are the conclusions drawn adequately supported by the results?

Partly

Are sufficient details of methods and analysis provided to allow replication by others?

Partly

Reviewer Expertise:

viral genomics, viral evolution

I confirm that I have read this submission and believe that I have an appropriate level of expertise to state that I do not consider it to be of an acceptable scientific standard, for reasons outlined above.

# Response

**Published:** 2024-09-05

**Authors:** Chong Han Ng

**Affiliations:** Multimedia University, Malaysia

In this manuscript, Dr Boon et al. analyzed the most common synonymous mutations of early pandemic SARS-CoV-2 genomes. They identified substitutions that influence the RNA secondary structure surrounding these positions. The authors have already modified and updated their manuscript based on previous comments, still there are few open questions in my opinion.

 First of all, it is clear that the dataset is not up to date and it will not be changed, still this should be clearly stated, starting from the title, reporting or the time period or something like "at the beginning of the pandemic''.

**Author response:**  The title has been revised to “Prediction of the effects of the top 10 synonymous mutations from 26645 SARS-CoV-2 genomes of early pandemic phase”.

**Reviewer comment:** Following this, also the introduction section should be focused on what we know about genomic variability and viral evolution in the time period being analyzed herein. If I understood well, most of these sequences belong to the alpha variant.

**Author response:** We added a few lines about Alpha variant in introduction, and result and discussion section (Identification of SARS-CoV-2 synonymous mutations).

**Reviewer comment:** Moreover, " variant of concern" (abbreviated VOC not VOI as stated in the text) refers to a specific lineage that carries several and distinct mutations. As it is stated in the text now it seems that a single mutation is a VOC. Please check it.

**Author response:** The abbreviation has been corrected from VOI to VOC. In the text, we mentioned that different SARS-CoV-2 variants with multiple synonymous and nonsynonymous mutations have been reported since the beginning of the outbreak. Therefore, it is clear that VOCs contain multiple mutations.

**Reviewer comment: **It is expected that most mutations are found in ORF1ab, as this covers most of the genome, so counts should be normalized by ORF length (e.g. fig.1).

**Author response:** Figure 1 show the distribution of 150 different types of synonymous mutations identified from 26645 SARS-CoV-2 genomes. It is not about the count or the average of the mutations in each ORFs, thus it should not be normalized.

**Reviewer comment: **Table 1 should report mutation frequency not count.

**Author response:** Table 1 shows the mutation frequency as reported in this paper.

**Reviewer comment:** Authors should discuss more why different predictors give very different results in terms of rna secondary structure, and how this can affect their analysis.

**Author response:** In the paper, there is about two paragraphs on comparison of these RNA secondary structure tools and we highlighted their similarities and differences of these tools. As mentioned in the discussion, we expected some differences in the outcomes obtained from different RNA secondary structure prediction tools due to the differences in algorithms, thermodynamic parameter settings, inclusion of pseudoknot calculation, incorporation of experimental data and the assumptions of each tool. Furthermore, SARS-CoV-2 may adopt different RNA secondary structure conformations. However, our major focus is to predict if the sSNP may affect/change RNA secondary structure, rather than the predicted structures *per se*. Importantly the outcomes allow us to prioritize variants for the experiment functional studies in the future. In addition, we demonstrated the usability of prediction tools by using 5’ UTR (1-480 nt) of SARS-CoV-2, which is well-characterized in other studies in the Extended data 1. For the four sSNPs which showed pronounced changes, we also compared the differences of these structures predicted by different tools.

**Reviewer comment:** Why did the author select 250 bp flanking positions for their prediction? Which is the rationale? How does this influence the results?

**Author response:** Some sSNPs affect only RNA secondary structures of neighboring nucleotides while some sSNPs may have long-ranging effects, in addition to the changes in the neighboring nucleotides. The length of RNA sequences used should not be too short since we may miss out the long-ranging effects. On the other hand, we need to bear in mind that most of RNA secondary structure prediction tools are not suitable for long sequences analysis. We tried out different lengths of flanking sequences, including 100b, 250b, 500b and 1000b, and we found out that 250b flanking sequences produce the optimal results.

**Reviewer comment:**  Are there any other mutations in the 501 nucleotide regions? Could mutations have an impact on predictions?

**Author response:** Yes, there may be other synonymous mutations within the 501 nucleotide regions, but they are not from the top 10 synonymous mutations. We are not doing epitasis analysis of different mutations since it is out of the scope of this study.

**Reviewer comment:** How did the authors handle overlapping/internal ORFs? In these situations a syn mutations could also be non-synonymous for the other ORF. I do not think this is the case for the top 10 mutations, but still this should be explained.

**Author response:** Yes, we don’t find any synonymous mutation of one ORF, which is also a synonymous mutation for another ORF. We included one sentence about the overlapping ORFs in the result and discussion section in Identification of SARS-CoV-2 synonymous mutation. Since some mutations, including C913U, U16176C are located near the boundaries of different ORFs, we postulated their potential long ranging effects at the neighboring ORFs in the discussion section.

**Reviewer comment:** The CUB section seems to me not really linked to the rest of the analyses and could be expanded more.

**Author response:** We expanded CUB section, including additional relevant references, elaborating the results further to make it clearer to the reader.

# Reviewer report: Reviewer response for version 2

**DOI:** 10.5256/f1000research.137703.r208617

**Published:** 2023-10-18

**Authors:** Roland Huber

**Affiliations:** 1 Bioinformatics Institute, A*STAR, Singapore

I agree with previous reviewers that the analyzed sequences represent only a limited sample of variation in SARS-CoV-2, specifically from early in the pandemic. This might introduce unexpected biases in the analysis. E.g. it would be more likely to observe host adaption early on which would be consistent with more favourable codon usage.

 With regard to the data that was analysed, the structure models obtained show limited consistency. The authors state that they do not expect the used tools to concur on the structures since they use different algorithms. This is concerning, as one would expect unambiguous structures to be consistent, even using different methodologies. Other tools, e.g. RNAstructure, also allow the inclusion of shape data and the prediction of pseudoknots. We are thus left with a series of diverging structure predictions and unsure what, if any, effect these specific mutations have. This is not helped by inconsistent presentation of the results. Figures 2-5 use different visualisations for the results of the 4 tools employed, which makes comparisons of the structures difficult for the reader.

 The study unfortunately does nothing to associate the regions or structural elements with any type of functional or biological information. We are thus left with a study that analyses a limited set of synonymous mutations using inconsistent structure predictions and offers no additional biological insight.

Is the work clearly and accurately presented and does it cite the current literature?

Partly

If applicable, is the statistical analysis and its interpretation appropriate?

Partly

Are all the source data underlying the results available to ensure full reproducibility?

Yes

Is the study design appropriate and is the work technically sound?

Partly

Are the conclusions drawn adequately supported by the results?

Partly

Are sufficient details of methods and analysis provided to allow replication by others?

Yes

Reviewer Expertise:

Computational Biology, Structural Genomics, RNA biology, Virology

I confirm that I have read this submission and believe that I have an appropriate level of expertise to state that I do not consider it to be of an acceptable scientific standard, for reasons outlined above.

# Reviewer report: Reviewer response for version 2

**DOI:** 10.5256/f1000research.137703.r149467

**Published:** 2022-09-06

**Authors:** Chandran Nithin

**Affiliations:** 1 Laboratory of Bioinformatics and Protein Engineering, International Institute of Molecular and Cell Biology in Warsaw, Warsaw, Poland

Authors have addressed the comments. However, the data set used in the study is outdated.

Is the work clearly and accurately presented and does it cite the current literature?

No

If applicable, is the statistical analysis and its interpretation appropriate?

Partly

Are all the source data underlying the results available to ensure full reproducibility?

Partly

Is the study design appropriate and is the work technically sound?

Partly

Are the conclusions drawn adequately supported by the results?

No

Are sufficient details of methods and analysis provided to allow replication by others?

Partly

Reviewer Expertise:

RNA structure prediction; Computational Structural Biology; RNA-protein complexes.

I confirm that I have read this submission and believe that I have an appropriate level of expertise to confirm that it is of an acceptable scientific standard.

# Reviewer report: Reviewer response for version 1

**DOI:** 10.5256/f1000research.76505.r135257

**Published:** 2022-04-28

**Authors:** Chandran Nithin

**Affiliations:** 1 Laboratory of Bioinformatics and Protein Engineering, International Institute of Molecular and Cell Biology in Warsaw, Warsaw, Poland

The manuscript titled "Prediction of the Effects of Synonymous Variants on SARS-CoV-2 Genome" analyzes 30,299 SARS-CoV-2 genomes retrieved from GISAID for the period between 31st December 2019 and 22nd March 2021. The study identifies 381 mutations in the genome, of which 150 are synonymous. The study discusses two mutations, C913U and C26735U.

1. The dataset used in this study is outdated and represents only a short period between 31st December 2019 and 22nd March 2021. It will be necessary to update the data to include more sequences from a wider time frame.
  The authors had indicated (through the editorial manager) that they are unwilling to update the dataset; the authors need to indicate the same in the title and detail the limitations of the dataset in the introduction section.
2. The authors have used the RNAfold program to predict the secondary structure of wild-type and mutant sequences. The authors need to use additional prediction tools and check for consensus between the predicted structure. Moreover, authors need to employ prediction tools to predict pseudoknots in the RNA secondary structure.
3. The secondary structure of SARS-CoV2 determined with the help of data from SHAPE-MaP, icSHAPE, and DMS-MaPseq experiments is available in the literature. ^1^, ^2^, ^3^ The authors should utilize this data to augment this study.
4. The study requires the inclusion of appropriate controls which quantify the effect of mutations on the stability of the RNA structure. The authors state that "The minimum free energy value of the mutant (- 146.90 kcal/mol) is slightly less negative than that of the wild type (- 147.40 kcal/mol), which makes it a less thermodynamically stable structure compared to the wild type." This claim needs to be explored in detail and supported with evidence from experimental data and literature.
5. The authors state that C26735U mutation induces changes in the predicted RNA secondary structure by forming an extra multibranch loop at the mutation site. The authors should compare the same with secondary systems mentioned in point 3 (See Ref 1, 2, and 3). The authors need to perform a similar analysis for C913U.
6. The study identifies 381 mutations in the genome, of which 150 are synonymous; however, it discusses only two. The authors need to provide the data on the remaining 148 synonymous mutations and briefly discuss some of them in the manuscript.
7. The authors need to expand and include relevant literature in the introduction section.
8. The manuscript needs to be edited for language errors.

Is the work clearly and accurately presented and does it cite the current literature?

No

If applicable, is the statistical analysis and its interpretation appropriate?

Partly

Are all the source data underlying the results available to ensure full reproducibility?

Partly

Is the study design appropriate and is the work technically sound?

Partly

Are the conclusions drawn adequately supported by the results?

No

Are sufficient details of methods and analysis provided to allow replication by others?

Partly

Reviewer Expertise:

RNA structure prediction; Computational Structural Biology; RNA-protein complexes.

I confirm that I have read this submission and believe that I have an appropriate level of expertise to state that I do not consider it to be of an acceptable scientific standard, for reasons outlined above.

## References

[1] : Secondary structural ensembles of the SARS-CoV-2 RNA genome in infected cells. *Nature Communications* .2022;13(1) : 10.1038/s41467-022-28603-2 10.1038/s41467-022-28603-2 PMC889130035236847

[2] : Comprehensive in vivo secondary structure of the SARS-CoV-2 genome reveals novel regulatory motifs and mechanisms. *Molecular Cell* .2021;81(3) : 10.1016/j.molcel.2020.12.041 584-598.e5 10.1016/j.molcel.2020.12.041 33444546 PMC7775661

[3] : Genome-wide mapping of SARS-CoV-2 RNA structures identifies therapeutically-relevant elements. *Nucleic Acids Research* .2020;48(22) : 10.1093/nar/gkaa1053 12436-12452 10.1093/nar/gkaa1053 PMC773678633166999

# Response

**Published:** 2022-08-22

**Authors:** Chong Han Ng

**Affiliations:** Multimedia University, Malaysia

The manuscript titled "Prediction of the Effects of Synonymous Variants on SARS-CoV-2 Genome" analyzes 30,299 SARS-CoV-2 genomes retrieved from GISAID for the period between 31st December 2019 and 22nd March 2021. The study identifies 381 mutations in the genome, of which 150 are synonymous. The study discusses two mutations, C913U and C26735U.

1. The dataset used in this study is outdated and represents only a short period between 31st December 2019 and 22nd March 2021. It will be necessary to update the data to include more sequences from a wider time frame.
  The authors had indicated (through the editorial manager) that they are unwilling to update the dataset; the authors need to indicate the same in the title and detail the limitations of the dataset in the introduction section.
**Response:** The landscape of mutational profile of SARS-CoV-2 genome is very dynamic, changing rapidly. As of 14 August 2022, there are more than 12 million SARS-CoV-2 virus genomes sequences deposited in GISAID database. Although it is useful to get more recent dataset, it is out of the scope of our study to update the data. Both identification of the SARS-CoV-2 mutations and the prediction analysis of the biological consequences of these mutations are very time-consuming processes. Based on the timelines of emergence of different variants, the cut-off date of our data collection overlaps with the period when the frequency of the alpha variant peaked. All the synonymous mutations except C241T reported in the alpha variant have been identified in our study as well. Although the alpha variant has been replaced by other variants in human population, it is detected from wildlife animals based on a few recent genomic surveillance analysis. It is possible that these alpha variant from wildlife animal may jump back to human, hence it remains relevant to predict the biological consequences of these synonymous mutations. The title of the paper has been revised accordingly. The limitation of the dataset was included in the discussion.
2. The authors have used the RNAfold program to predict the secondary structure of wild-type and mutant sequences. The authors need to use additional prediction tools and check for consensus between the predicted structure. Moreover, authors need to employ prediction tools to predict pseudoknots in the RNA secondary structure.
**Response: **To improve the outcome of the RNA secondary structure analysis, 3 prediction tools, namely RNAfold, IPknot++ and MXfold2 are used in our study. Of these, IPknot++ predicted some synonymous mutations may affect the pseudoknot formation. The prediction results for all top 10 synonymous mutations using these 3 tools are summarized in the Table 2
3. The secondary structure of SARS-CoV2 determined with the help of data from SHAPE-MaP, icSHAPE, and DMS-MaPseq experiments is available in the literature.1,2,3 The authors should utilize this data to augment this study
**Response: **To improve the outcome of the prediction analysis, the RNA secondary structure of wild type and mutant sequences were predicted using RNAfold program with the incorporation of SHAPE reactivity data obtained from the study done by Manfredonia *et al.* (2020).
4. The study requires the inclusion of appropriate controls which quantify the effect of mutations on the stability of the RNA structure. The authors state that "The minimum free energy value of the mutant (- 146.90 kcal/mol) is slightly less negative than that of the wild type (- 147.40 kcal/mol), which makes it a less thermodynamically stable structure compared to the wild type." This claim needs to be explored in detail and supported with evidence from experimental data and literature.
**Response: **Instead of comparing minimum free energy between the wild type and mutant, we applied three RNA secondary structure prediction tools to predict the effect of the mutation on RNA secondary structure.
5. The authors state that C26735U mutation induces changes in the predicted RNA secondary structure by forming an extra multibranch loop at the mutation site. The authors should compare the same with secondary systems mentioned in point 3 (See Ref 1, 2, and 3). The authors need to perform a similar analysis for C913U.
**Response: **The results of the 4 mutations showing changes in all 3 prediction tools are included in Figure 2-5.  
6. The study identifies 381 mutations in the genome, of which 150 are synonymous; however, it discusses only two. The authors need to provide the data on the remaining 148 synonymous mutations and briefly discuss some of them in the manuscript.
**Response: **The RNA secondary structures prediction results using 3 prediction tools for the top 10 synonymous mutations have been included in Extended data 1-3. There were 4 mutations showing changes in all 3 prediction tools and the results are discussed in the manuscript.
7. The authors need to expand and include relevant literature in the introduction section.
**Response: **The relevant literature in the introduction section has been updated.
8. The manuscript needs to be edited for language errors.
**Response: **The language errors have been edited accordingly.

# Reviewer report: Reviewer response for version 1

**DOI:** 10.5256/f1000research.76505.r101828

**Published:** 2021-12-20

**Authors:** Leyi Wang

**Affiliations:** 1 Veterinary Diagnostic Laboratory and Department of Veterinary Clinical Medicine, College of Veterinary Medicine, University of Illinois, Urbana, IL, USA

The manuscript entitled “Prediction of the Effects of Synonymous Variants on SARS-CoV-2 Genome” reports an analysis of the SARS-CoV-2 genome obtained in the GISAID database. The authors analyzed synonymous changes of the 30,229 SARS-CoV-2 sequences available online, and the effects of these changes on RNA secondary structures and base-pair probability. They identified 150 synonymous mutations in 11 coding regions. Mutations with higher frequency are mostly found in ORF1a and ORF1b and two mutations (C913U and C26735U) found with effect on the predicted RNA secondary structure are not those with higher frequency.

 This study collected 30,229 sequences in the database in the period of 31 Dec 2019 to 22 March 2021. As of 17 Dec 2021, there are over 4.4 million complete genomes with high coverage. Will these sequences used in the study include all variants reported or what sequences of variants are included? A table to summarize all variants with these mutations or not is needed.

 A major concern is that the sequences even filtered by setting parameters to keep only sequencing with complete genome and high coverage still contain bad sequences, how did the authors exclude those sequences?

 In the abstract section, the results part only included those mutations with high frequency but did not contain results of other analyses. Instead, they included the results of RNA secondary structure analysis in the conclusion.

Is the work clearly and accurately presented and does it cite the current literature?

Partly

If applicable, is the statistical analysis and its interpretation appropriate?

Yes

Are all the source data underlying the results available to ensure full reproducibility?

Yes

Is the study design appropriate and is the work technically sound?

Partly

Are the conclusions drawn adequately supported by the results?

Partly

Are sufficient details of methods and analysis provided to allow replication by others?

Yes

Reviewer Expertise:

Clinical Virology Diagnosis

I confirm that I have read this submission and believe that I have an appropriate level of expertise to confirm that it is of an acceptable scientific standard, however I have significant reservations, as outlined above.

# Response

**Published:** 2022-08-22

**Authors:** Chong Han Ng

**Affiliations:** Multimedia University, Malaysia

The manuscript entitled “Prediction of the Effects of Synonymous Variants on SARS-CoV-2 Genome” reports an analysis of the SARS-CoV-2 genome obtained in the GISAID database. The authors analyzed synonymous changes of the 30,229 SARS-CoV-2 sequences available online, and the effects of these changes on RNA secondary structures and base-pair probability. They identified 150 synonymous mutations in 11 coding regions. Mutations with higher frequency are mostly found in ORF1a and ORF1b and two mutations (C913U and C26735U) found with effect on the predicted RNA secondary structure are not those with higher frequency.

 This study collected 30,229 sequences in the database in the period of 31 Dec 2019 to 22 March 2021. As of 17 Dec 2021, there are over 4.4 million complete genomes with high coverage. Will these sequences used in the study include all variants reported or what sequences of variants are included? A table to summarize all variants with these mutations or not is needed.

**Response: **We downloaded the raw data on 23 ^rd^ March 2021 and completed the data analysis in late June 2021. We submitted the manuscript in late August 2021. The landscape of mutational profile of SARS-CoV-2 genome is very dynamic, changing rapidly. It is far beyond the scope of this study to include all variants reported. Based on the timelines of emergence of different variants, the cut-off date of our data collection overlaps with the period when the frequency of the alpha variant peaked. All the synonymous mutations except C241T reported in the alpha strain have been identified in our study as well.  We added new paragraph in the discussion section to discuss the impact of our findings and to explain why the study of alpha variant remains relevant now. In addition, we used 3 RNA secondary prediction analysis tool, instead of one, to improve the outcome of the analysis. The prediction results for all top 10 synonymous mutations using these 3 tools and the base pair probability estimation results are summarized in the Table 2.

 A major concern is that the sequences even filtered by setting parameters to keep only sequencing with complete genome and high coverage still contain bad sequences, how did the authors exclude those sequences?

**Response: **In our original method, we used the high coverage filter when downloading from GISAID database, in which only entries with less 1% N and 0.05% unique amino acids mutations are included. To further reduce bad sequences, we filtered to remove those sequences with higher than 0.1% N unresolved nucleotides and ambiguous letters. A total of 3584 sequences were removed by applying this filter. The list of the synonymous mutations remains unchanged despite of the revision of the mutation frequency.

 In the abstract section, the results part only included those mutations with high frequency but did not contain results of other analyses. Instead, they included the results of RNA secondary structure analysis in the conclusion.

**Response**: The abstract has been revised to update the results and conclusion.

# Reviewer report: Reviewer response for version 1

**DOI:** 10.5256/f1000research.76505.r97299

**Published:** 2021-11-02

**Authors:** Takahiko Koyama

**Affiliations:** 1 IBM TJ Watson Research Center, Yorktown Heights, NY, USA

Boon *et al.* made an investigation of synonymous mutations of SARS-CoV-2 genomes. Authors first identified common synonymous mutations such as 3037C>T followed by secondary structure predictions using RNAfold. 

 First, the number of genomes authors used is too small and too old. Currently, nearly 5 million genomes are uploaded at GISAID; however, authors used only 30,229. The set does not represent the current mutational landscape of SARS-CoV-2. Authors need to update the data. I have 3 million genomes analyzed and 18108 distinct synonymous mutations identified. The list of highest frequency in Table 1 looks completely different with the latest dataset.

 Secondly, authors employed RNAfold for RNA secondary structure prediction. Although RNAfold is a standard software for the task, the accuracy of the prediction is not impressive. Authors need to explore various other tools for the secondary structure or justify the usage of RNAfold.

Is the work clearly and accurately presented and does it cite the current literature?

Partly

If applicable, is the statistical analysis and its interpretation appropriate?

Yes

Are all the source data underlying the results available to ensure full reproducibility?

Yes

Is the study design appropriate and is the work technically sound?

Partly

Are the conclusions drawn adequately supported by the results?

Partly

Are sufficient details of methods and analysis provided to allow replication by others?

Yes

Reviewer Expertise:

Genome Analysis, SARS-CoV-2, Cancer, Immunology, Stem cell

I confirm that I have read this submission and believe that I have an appropriate level of expertise to state that I do not consider it to be of an acceptable scientific standard, for reasons outlined above.

# Response

**Published:** 2021-11-04

**Authors:** Chong Han Ng

**Affiliations:** Multimedia University, Malaysia

Boon et al. made an investigation of synonymous mutations of SARS-CoV-2 genomes. Authors first identified common synonymous mutations such as 3037C>T followed by secondary structure predictions using RNAfold.

 First, the number of genomes authors used is too small and too old. Currently, nearly 5 million genomes are uploaded at GISAID; however, authors used only 30,229. The set does not represent the current mutational landscape of SARS-CoV-2. Authors need to update the data. I have 3 million genomes analyzed and 18108 distinct synonymous mutations identified. The list of highest frequency in Table 1 looks completely different with the latest dataset.

 RE: Thank you for the comments and feedbacks. We downloaded SARS-CoV-2 virus genome data submitted from 31 December 19 to 22 March 21 on 23 March 21. There were about a total of 842,603 SARS-CoV-2 virus genomes sequences before applying filters. SARS-CoV-2 genomic sequences were filtered by setting parameters to keep only sequences with complete genome, high coverage, low coverage excluded and patient status options. The use of the low coverage excluded and patient status filters were unintentionally left out in the manuscript and they will be added later. In our study, the use of these filters allows us to analyze high quality data to identify SARS-CoV-2 mutations without the requirement of high computing power. Our data is representative of the mutational landscape of SARS-CoV-2 for this specified period. Same synonymous mutations, including C913T, C3037T, C5986T, C14676T, C15279T and T16176C were identified in alpha strain by other research groups ( https://covariants.org/variants/20I.Alpha.V1) and ( https://assets.publishing.service.gov.uk/government/uploads/system/uploads/attachment_data/file/959438/Technical_Briefing_VOC_SH_NJL2_SH2.pdf). The frequency of alpha strain peaked in April-May 21 and subsequently it has been quickly replaced by delta strain. Interestingly the defining synonymous mutations of delta strain ( https://covariants.org/variants/21A.Delta) are very different from those of alpha strain. The mutational landscape of SARS-CoV-2 genome is very dynamic as shown by other research groups. Bear in mind that the main objective of our paper is not to monitor the mutational changes of SARS-CoV-2 genome. Although it is useful to get more recent dataset, it is out of the scope of our study to update the data. Both identification of the SARS-CoV-2 mutations and the prediction analysis of the biological consequences of these mutations are very time-consuming processes. The first draft of the manuscript was prepared in late June 21 and submitted in late August 21. Since different SARS-CoV-2 variants may have different sets of mutations, we will gain more insight to study the effects of different mutations in different SARS-CoV-2 variants. In our follow-up study, we plan to predict and compare the effect of the mutations of different variants. These analyses may provide some plausible explanation why some variants are replaced by other variants eventually.

 Secondly, authors employed RNAfold for RNA secondary structure prediction. Although RNAfold is a standard software for the task, the accuracy of the prediction is not impressive. Authors need to explore various other tools for the secondary structure or justify the usage of RNAfold.

 RE: We chose RNAfold because it is the most commonly used tools to predict the RNA secondary structure. Other than RNAfold, we also considered using Mfold to perform the RNA secondary structure prediction as well. However, the Mfold server was down during the time of the study. RNAfold computes an optimal structure over the whole sequence length, which means its performance and accuracy can be affected as the sequences used get longer. However, in our case, the accuracy of the prediction is still acceptable since the sequence we used to do the structure prediction is relatively short. Besides, the prediction results we obtained from RNAfold is further supported with the prediction results obtained from MutaRNA, which predicts the structural changes induced by the mutation by estimating the base pairing probabilities. In our results, the circular plot from MutaRNA shows the changes in the base pairing probabilities near the mutation site correlates well with the RNA secondary structure predicted by RNAfold.

# Response

**Published:** 2022-11-21

**Authors:** Chong Han Ng

**Affiliations:** Multimedia University, Malaysia

First, the number of genomes authors used is too small and too old. Currently, nearly 5 million genomes are uploaded at GISAID; however, authors used only 30,229. The set does not represent the current mutational landscape of SARS-CoV-2. Authors need to update the data. I have 3 million genomes analyzed and 18108 distinct synonymous mutations identified. The list of highest frequency in Table 1 looks completely different with the latest dataset.

**RE:** We submitted a revised version in Sep 2022. Although we didn’t use the latest dataset, we provide some justification in the discussion to explain why our data may remain relevant. We hope you will consider to review our manuscript again. The landscape of mutational profile of SARS-CoV-2 genome is very dynamic, changing rapidly. It is far beyond the scope of this study to include all variants reported. Based on the timelines of emergence of different variants, the cut-off date of our data collection overlaps with the period when the frequency of the alpha variant peaked. All the synonymous mutations except C241T reported in the alpha strain have been identified in our study as well. Although the alpha variant has been replaced by other variants in human population, it is detected from wildlife animals based on a few recent genomic surveillance analysis. It is possible that these alpha variant from wildlife animal may jump back to human, hence it remains relevant to predict the biological consequences of these synonymous mutations. The title of the paper has been revised accordingly. The limitation of the dataset was included in the discussion.

 Secondly, authors employed RNAfold for RNA secondary structure prediction. Although RNAfold is a standard software for the task, the accuracy of the prediction is not impressive. Authors need to explore various other tools for the secondary structure or justify the usage of RNAfold.

**RE:** In the revised version, we used 3 RNA secondary prediction analysis tool, namely RNAfold, IPknot++, to improve the outcome of the analysis. The prediction results for all top 10 synonymous mutations using these 3 tools and the base pair probability estimation results are summarized in the Table 2.
